# *k*-Resolved Ultrafast Light-Induced Band Renormalization in Monolayer WS_2_ on Graphene

**DOI:** 10.1021/acs.nanolett.4c06238

**Published:** 2025-01-08

**Authors:** Niklas Hofmann, Alexander Steinhoff, Razvan Krause, Neeraj Mishra, Giorgio Orlandini, Stiven Forti, Camilla Coletti, Tim O. Wehling, Isabella Gierz

**Affiliations:** † Institute for Experimental and Applied Physics, University of Regensburg, 93040 Regensburg, Germany; ‡ Institute for Theoretical Physics, Universität Bremen, P.O. Box 330 440, 28334 Bremen, Germany; § Bremen Center for Computational Materials Science, Universität Bremen, 28334 Bremen, Germany; ∥ Center for Nanotechnology Innovation@NEST, Istituto Italiano di Tecnologia, 56127 Pisa, Italy; ⊥ Graphene Laboratories, Istituto Italiano di Tecnologia, 16163 Genova, Italy; # I. Institute of Theoretical Physics, University of Hamburg, Notkestrasse 9, 22607 Hamburg, Germany; ∇ The Hamburg Centre for Ultrafast Imaging, Luruper Chaussee 149, 22761 Hamburg, Germany

**Keywords:** monolayer transition metal dichalcogenides, time- and angle-resolved photoemission spectroscopy, band gap renormalization, dielectric screening, ab initio calculations, nonequilibrium Green functions

## Abstract

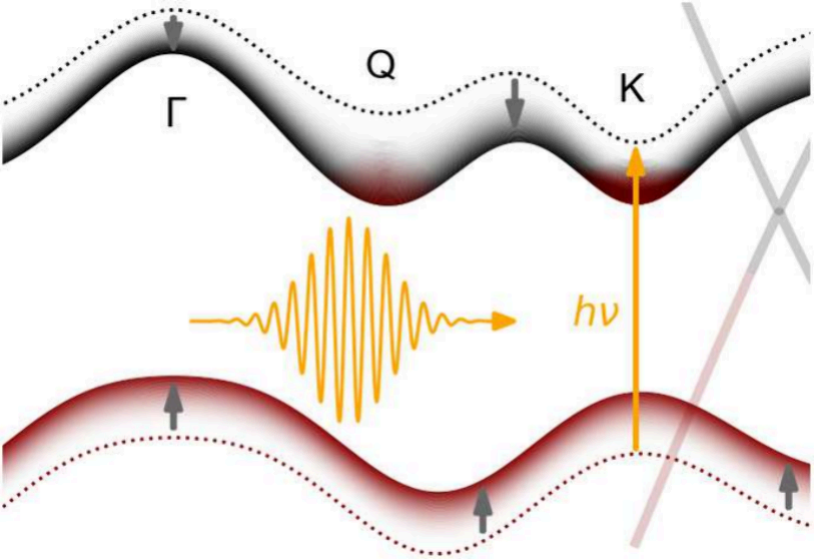

Understanding and controlling the electronic properties of two-dimensional materials are crucial for their potential applications in nano- and optoelectronics. Monolayer transition metal dichalcogenides have garnered significant interest due to their strong light–matter interaction and extreme sensitivity of the band structure to the presence of photogenerated electron–hole pairs. In this study, we investigate the transient electronic structure of monolayer WS_2_ on a graphene substrate after resonant excitation of the A-exciton using time- and angle-resolved photoemission spectroscopy. We observe a pronounced band structure renormalization, including a substantial reduction of the transient band gap in good quantitative agreement with our *ab initio* theory, revealing the importance of both intrinsic WS_2_ and extrinsic substrate contributions. Our findings deepen the fundamental understanding of band structure dynamics in two-dimensional materials and offer valuable insights for the development of novel electronic and optoelectronic devices based on monolayer TMDs and their heterostructures with graphene.

The size of the band gap of a semiconductor is the essential parameter that determines its optical and electronic properties, with crucial importance for light absorption in solar cells and photodetectors as well as the performance of transistors and other electronic devices. In two-dimensional (2D) semiconductors such as monolayer transition metal dichalcogenides (TMDs), confinement and reduced screening give rise to strong electronic correlations that modify the size of the quasi-particle gap and lead to the formation of excitons with large binding energies that dominate the optical response. Controlling the band gap of 2D semiconductors is highly relevant for the development of next generation optoelectronic devices. This is commonly done by tailoring dielectric screening. At equilibrium, this has been achieved by embedding 2D semiconductors in van der Waals (vdW) heterostructures^1,2^ or by changing the carrier density with a gate voltage.^3^ Out of equilibrium, photodoping with femtosecond laser pulses has been used to modify the screening dynamically, which offers the possibility to control the size of the band gap on femtosecond time scales.^4−12^

*Ab initio* theory predicts that the band shifts caused by the presence of excited carriers are nonrigid with a pronounced momentum dependence.^13,14^ To date, however, experiments have mainly probed the transient size of the direct gap at the K-point. A possible momentum dependence of the transient gap remains unexplored.

Here, we use time- and angle-resolved photoemission spectroscopy (trARPES) to probe the transient band structure of a 2D semiconductor over the entire Brillouin zone. For this purpose, we use monolayer WS_2_ on a graphene/SiC(0001) substrate that we excite at resonance to the A-exciton at *ℏω*_pump_ = 2 eV. We extract the size of the direct quasiparticle gap at the K-point as well as the momentum-resolved valence band (VB) shift as a function of time. The direct gap is found to shrink by Δ*E* ∼ 140 meV for a fluence of 1.5 mJ cm^–2^. Within the experimental error bars, the transient VB shift is found to be rigid between Γ and K with a fluence-dependent amplitude ranging from Δ*E* ∼ 100 meV to Δ*E* ∼ 170 meV for fluences between 0.7 mJ cm^–2^ and 1.5 mJ cm^–2^. Our trARPES results further provide access to the transient carrier distribution and temperature that serve as input parameters for advanced *ab initio* calculations that include the Hartree and GW contributions of WS_2_ as well as GdW contributions of the graphene substrate. The calculations quantitatively reproduce the experimentally observed shifts. In addition, our theory allows us to disentangle the relevance of the individual contributions and to determine the momentum dependence of the band shifts.

The detailed microscopic understanding gained in this work provides important information for the design of next generation optoelectronic devices.

## Sample Growth

4H-SiC substrates were H-etched to remove scratches and subsequently graphitized in an Ar atmosphere. The resulting carbon monolayer with R30° structure was decoupled from the SiC substrate by H-intercalation, yielding quasi-freestanding monolayer graphene on H-terminated SiC(0001).^15^ WS_2_ was then grown by chemical vapor deposition from solid WO_3_ and S precursors.^16^ Atomic force microscopy (AFM) and secondary electron microscopy (SEM) revealed that WS_2_ grows in the shape of triangular islands with side lengths in the range of 300–700 nm with well-defined twist angles of either 0° or 30° with respect to the graphene layer.^12^

## trARPES

The setup was based on a commercial titanium sapphire amplifier (Astrella, Coherent) with a central wavelength of 800 nm, a repetition rate of 1 kHz, a pulse duration of 35 fs, and a pulse energy of 7 mJ. Five mJ was used to seed a commercial optical parametric amplifier (Topas Twins, Light Conversion), the signal output of which was frequency doubled, yielding 2 eV pump pulses resonant with the A-exciton of monolayer WS_2_. The remaining 2 mJ of output energy was frequency doubled and focused onto an argon gas jet for high harmonic generation. A single harmonic at 21.7 eV photon energy was selected with a grating monochromator, yielding extreme ultraviolet (XUV) probe pulses that were used to eject photoelectrons from the sample. The photoelectrons were dispersed according to their kinetic energy and emission angle by a hemispherical analyzer (Phoibos 100, SPECS), yielding 2D snapshots of the occupied part of the band structure in momentum space. The probe spot diameter was ∼250 μm on the sample, covering many different WS_2_ islands. Nevertheless, 0° and 30° WS_2_ islands were easily distinguished based on the dispersion of their band structure in momentum space. The energy and temporal resolutions for the measurements presented in this publication were ∼200 meV and 160 fs, respectively.

## Theory

We combine nonequilibrium Green functions with *ab initio* calculations of the ground state properties to compute the influence of photoexcited electron–hole pairs on the transient electronic structure of monolayer WS_2_ on a graphene/SiC substrate across the whole Brillouin zone. Electrons and holes are assumed to follow a quasithermal distribution with one common elevated temperature. The influence of excited carriers inside the WS_2_ layer is treated explicitly in the GW self-energy. The contribution of excited carriers inside the graphene layer enters via a macroscopic dielectric function. We consider both Hartree and static as well as dynamic exchange renormalizations. Further details are provided in the Supporting Information (SI).

**Figure 1:**
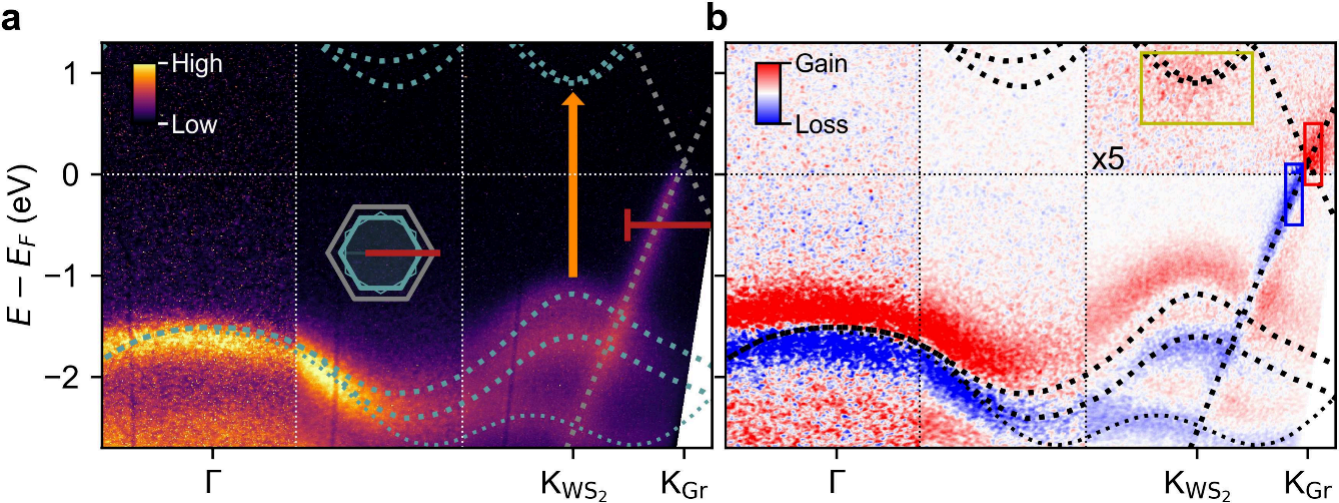
**trARPES data of the WS**_2_**-graphene heterostructure. (a)** ARPES spectrum measured at negative pump–probe delay before the arrival of the pump pulse along the ΓK direction as indicated by the red line in the inset. The orange vertical arrow illustrates the excitation resonant to the A-exciton at *ℏω*_pump_ = 2.0 eV. Gray and green dashed lines indicate the theory band structures for graphene^18^ and WS_2_,^17^ respectively, that were shifted in energy to match the observed band alignment. The thin dashed green line marks the band structure of WS_2_ flakes with 30° rotation relative to the graphene layer. The horizontal red line covers the *k* range over which EDCs in Figure 3b are extracted. **(b)** Pump-induced changes 250 fs after excitation at *ℏω*_pump_ = 2.0 eV with a fluence of 1.5 mJ cm^2^. Red and blue indicate a gain and loss of photocurrent with respect to negative pump–probe delays, respectively. The upper right panel is multiplied with a factor of 5 for better visibility. Colored boxes indicate the area of integration for the pump–probe traces in Figure 3a.

Figure 1a shows the band structure of the WS_2_-graphene sample measured along the ΓK-direction of the 0° WS_2_ islands at a negative pump–probe delay before the arrival of the pump pulse. Gray and green dashed lines are theoretical band structures from ref (17) for monolayer WS_2_ and from ref (18) for monolayer graphene that have been shifted in energy to account for the experimentally observed doping and equilibrium gap size. The thin dashed green line indicates the dispersion of the 30° WS_2_ islands. The orange arrow highlights the direct electronic transition triggered by photoexcitation at *ℏω*_pump_ = 2 eV. Figure 1b shows the pump-induced changes in the photocurrent at a pump–probe delay of *t* = 250 fs for a pump fluence of *F* = 1.5 mJ cm^–2^. Red and blue indicate gain and loss, respectively, with respect to the unperturbed photocurrent in Figure 1a. The pump–probe signal exhibits three main features: (1) The minimum of the WS_2_ conduction band (CB) at K_WS_2__ gets populated by the pump pulse. (2) The WS_2_ VB exhibits a loss at its equilibrium position and a gain above, indicative of a transient upshift. Note that the loss at the equilibrium position of the upper valence band is largely compensated by the upshift of the lower valence band. (3) The Dirac cone of graphene shows a loss of photoelectrons below and a gain of photoelectrons above the Fermi level, suggestive of a hot Fermi–Dirac distribution.

**Figure 2:**
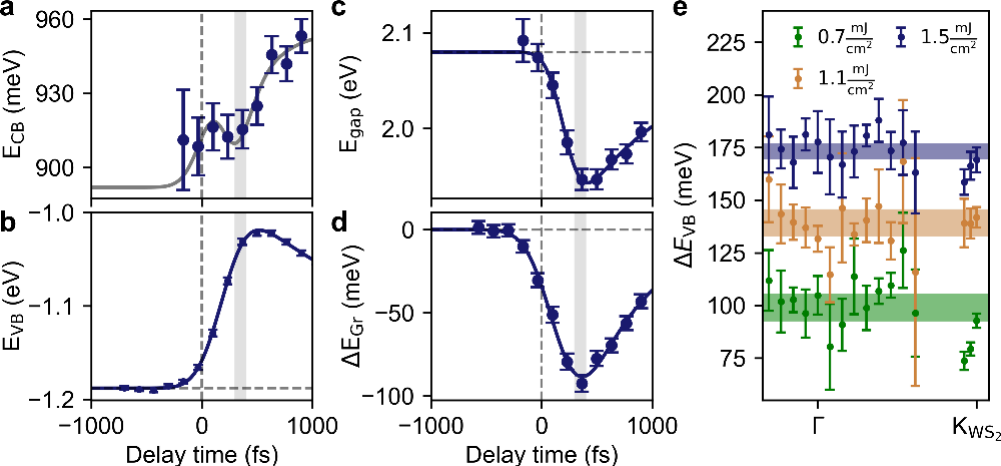
**trARPES data analysis. (a)** Transient CB position. The gray line is a guide to the eye obtained by adding the transient band gap fit from panel c to the fit of the VB position from panel b. **(b)** Transient band shift of the upper VB at the K point together with exponential decay fit. **(c)** Transient band gap obtained by subtracting the data from panels a and b together with exponential decay fit. **(d)** Graphene band shift together with exponential decay fit. Vertical gray lines in panels a–d mark the pump–probe delay where the VB shift at K reaches its maximum. **(e)***k*-dependent VB shifts for different excitation fluences for the pump–probe delay where the VB shift at K reaches its maximum. Horizontal lines represent momentum-averaged shifts. The line width reflects the standard deviation. The error bars in panels a, b, d, and e represent the standard deviation determined from the data fitting. The error bars in panel c are the sum of the error bars in panels a and b.

In order to extract the transient band gap at K_WS_2__ and the momentum-resolved VB shift, we proceed as follows: we extract energy distribution curves (EDCs) at different momenta that we fit with an appropriate number of Gaussian peaks and a Shirley background to determine the transient binding energy of the WS_2_ VB and CB and the Dirac cone of graphene. Further details are provided in the SI. The transient peak positions for the WS_2_ CB and VB at K_WS_2__ are shown in Figures 2a and b, respectively. The transient band gap obtained by subtracting the binding energy of the WS_2_ VB in Figure 2b from the binding energy of the WS_2_ CB in Figure 2a is shown in Figure 2c together with an exponential fit (see the SI). The band gap is found to decrease by Δ*E*_gap_ = 140 ± 20 meV with a lifetime of τ = 0.9 ± 0.2 ps in good agreement with our own previous results^9^ and slightly lower than typical experimental^4,6,8^ and theoretical values^19−22^ reported in the literature for similar samples. Possible reasons for this minor discrepancy might be related to the use of different substrates and the difficulty in estimating the density of photoexcited electron–hole pairs in the experiment (see below). Figure 2d shows the transient binding energy of the Dirac cone together with an exponential fit (see the SI). The Dirac cone is found to shift by Δ*E* = 90 ± 10 meV with a lifetime of τ = 0.6 ± 0.1 ps. The momentum-resolved shift of the WS_2_ VB is shown in Figure 2e for a pump–probe delay of *t*_max_ ∼ 500 fs, where the VB shift at K_WS_2__ reaches its maximum for three different fluences. Within the error bars, the observed VB shift is found to be momentum-independent with amplitudes of Δ*E* = 100 ± 10 meV, Δ*E* = 140 ± 10 meV, and Δ*E* = 170 ± 10 meV for fluences of *F* = 0.7 mJ cm^–2^, *F* = 1.0 mJ cm^–2^, and *F* = 1.5 mJ cm^–2^, respectively. This is similar to the previously observed rigid band shift for monolayer WS_2_ resting on different substrates^2^ and consistent with previous predictions of *ab initio* theory for photoexcited samples.^13,14^

**Figure 3:**
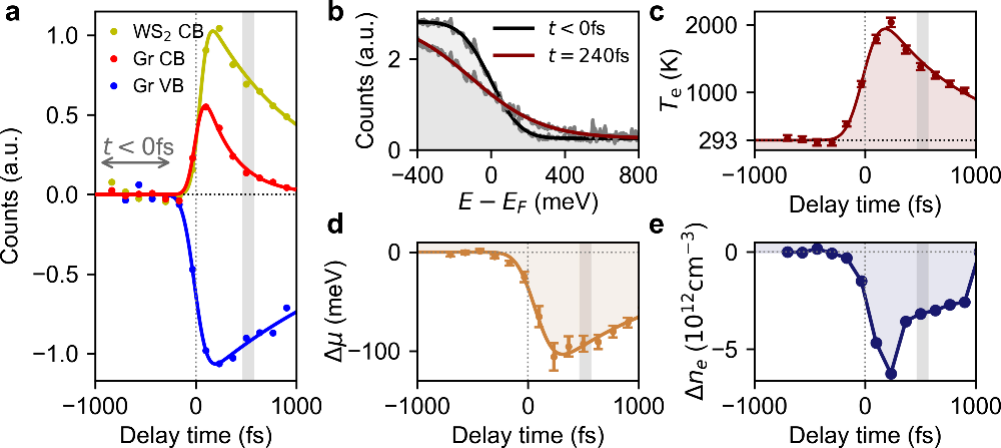
**Charge transfer dynamics. (a)** Photocurrent integrated over the colored boxes from Figure 1b. The gray arrow marks the integration range for the ARPES spectrum in Figure 1a. **(b)** Energy distribution curve showing the Fermi edge in the Dirac cone for two different pump–probe delays together with Fermi–Dirac fits. **(c)** Electronic temperature in the Dirac cone as a function of pump–probe delay together with exponential decay fit. **(d)** Chemical potential inside the graphene layer as a function of pump–probe delay together with exponential decay fit. **(e)** Changes in carrier density in the graphene layer, calculated from the electronic temperature from panel c, the chemical potential from panel d, and the density of states. Vertical gray lines in panels a, c, d, and e mark the pump–probe delay where the VB shift at K reaches its maximum. The error bars in panels d and e represent the standard deviation determined from the data fitting.

Next, we determine the nonequilibrium carrier distribution of the WS_2_-graphene sample at the pump–probe delay that corresponds to the momentum-resolved WS_2_ VB shift shown in Figure 2e to provide input for subsequent theory. Figure 3a shows the photocurrent integrated over the three areas marked by colored boxes in Figure 1b as a function of pump–probe delay together with exponential fits. The Dirac cone of graphene shows a short-lived gain (red, τ = 300 ± 30 fs) and a long-lived loss (blue, τ = 2.10 ± 0.03 ps). The lifetime of the electrons at the bottom of the WS_2_ CB (yellow) is found to be τ = 950 ± 70 fs. Figure 3b shows the energy-resolved population of the Dirac cone, obtained by integrating the photocurrent over the momentum range indicated by the red scale bar in Figure 1a, for two different time delays together with Fermi–Dirac fits (see the SI). The resulting electronic temperature and chemical potential are shown in Figures 3c and d, respectively. The electronic temperature reaches a peak value of *T*_e,max_ = 1900 ± 100 K and cools with an exponential lifetime of τ = 760 ± 60 fs. From the electronic temperature and the chemical potential, we calculate the carrier concentration inside the Dirac cone as explained in detail in the SI. The result is shown in Figure 3e as a function of the pump–probe delay. We find that the carrier concentration inside the Dirac cone transiently decreases by (5.7 ± 1.2) × 10^12^ cm^–2^.

The observations in Figures 2 and 3 have been previously attributed to ultrafast charge separation in WS_2_-graphene heterostructures.^9,12,23^ Photoexcitation at resonance to the A-exciton of WS_2_ is followed by rapid hole transfer into the graphene layer, resulting in a charge-separated transient state with a lifetime of ∼1 ps. For a pump–probe delay of *t* ∼ 500 fs, where the VB shift at K_WS_2__ reaches its maximum, we find the following carrier distribution in our WS_2_-graphene sample: ∼3.4 × 10^12^ cm^–2^ holes are transferred from WS_2_ to graphene, while ∼70% of the photoexcited electrons remain in the WS_2_ layer. The electrons inside the Dirac cone exhibit an elevated electronic temperature of *T*_e_ ∼ 1400 K. The values for all fluences investigated in the present work are summarized in Table 1.

**Table 1:** Time Delay *t*_max_, Where the WS_2_ VB Shift at K Reaches Its Maximum; Hole Density inside the Dirac Cone *n*_h_^gr^(*t* = *t*_max_); Electronic Temperature of Carriers inside the Dirac Cone *T*_e_(*t* = *t*_max_)

pump fluence	*t*_max_	*n*_h_^gr^	*T*_e_
0.7 mJ cm^–2^	450 fs	7.9 × 10^12^ cm^–2^	1000 K
1.0 mJ cm^–2^	490 fs	8.8 × 10^12^ cm^–2^	1300 K
1.5 mJ cm^–2^	520 fs	10.4 × 10^12^ cm^–2^	1400 K

In Table 2 we present our estimates for the total density of photogenerated electron–hole pairs  for the three pump fluences employed in the experiment. This quantity is difficult to estimate as the absorption of 2D TMDs is highly nonlinear due to Pauli blocking and many-body effects.^14^ According to ref (14),  for hBN-encapsulated WS_2_ is predicted to saturate around 0.5 × 10^14^ cm^–2^ for fluences ≥0.5 mJ cm^–2^. A lower limit for  is given by the maximum number of holes that are found to be transferred into the graphene layer during ultrafast charge separation.

**Table 2:** Estimated Upper and Lower Bound for the Density of Photo-Generated Electron–Hole Pairs for Different Fluences

pump fluence	upper limit	lower limit
0.7 mJ cm^–2^	5 × 10^13^ cm^–2^	2 × 10^12^ cm^–2^
1.0 mJ cm^–2^	5 × 10^13^ cm^–2^	3 × 10^12^ cm^–2^
1.5 mJ cm^–2^	5 × 10^13^ cm^–2^	6 × 10^12^ cm^–2^

**Figure 4:**
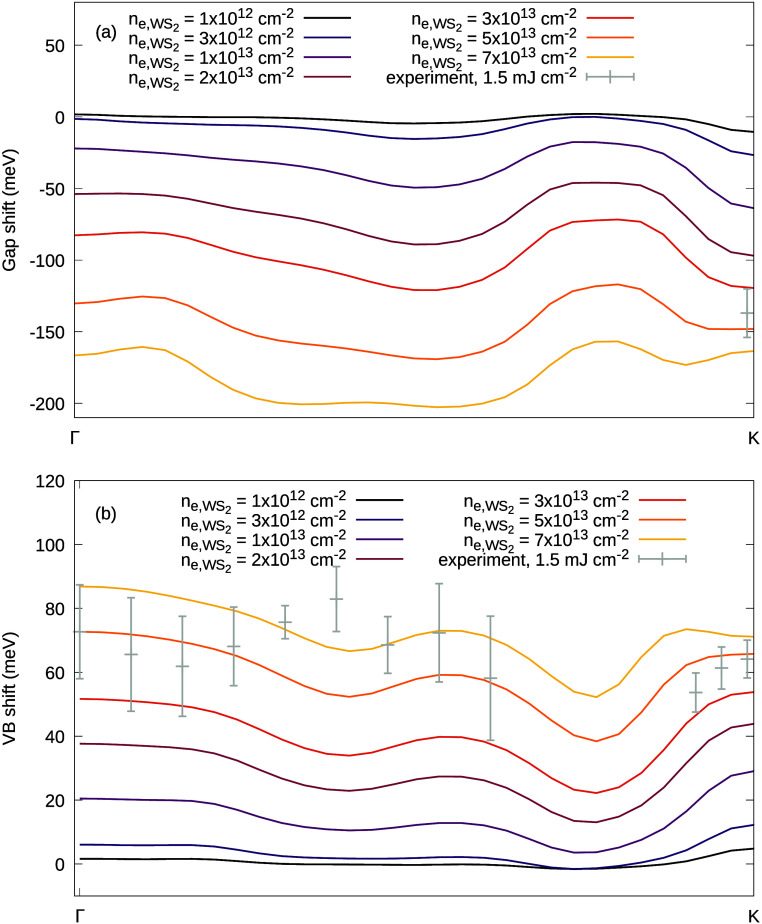
**Theory.** Calculated *k*-resolved transient band gap (a) and quasi-particle shifts of WS_2_ VB (b) for different carrier densities for a carrier temperature of *T* = 1500 K. The gray dots represent charging-shift-corrected experimental data points for a fluence of 1.5 mJ cm^–2^.

The parameters in Tables 1 and 2 now serve as inputs for *ab initio* calculations of the transient band gap renormalization and WS_2_ VB shift. At equilibrium, the graphene layer is found to be hole-doped with the Fermi level at −300 meV below the Dirac point (see Figure 1a) corresponding to a hole concentration of *n*_h_^gr,0^ = 7 × 10^12^ cm^–2^. First, we correct the band structure of freestanding monolayer WS_2_ by adding static GdW corrections due to screening from the graphene/SiC substrate. Next, we computed the transient changes of the WS_2_ band structure due to screening from the photoexcited electron–hole pairs. For this purpose we assume initial photoexcited electron and hole densities in the range between  cm^–2^ and  cm^–2^ corresponding to the estimates provided in Table 2. Further, 90% of the photoexcited holes are assumed to be transferred into the graphene layer.^24^ For a WS_2_ coverage of the graphene/SiC substrate of 50%^12^ this corresponds to a hole density of  inside the graphene layer. Finally, we assume that all carriers have one common electronic temperature in the range between *T* = 1000 K and *T* = 1400 K (see Table 1) irrespective of their nature (electron or hole) and their location (WS_2_ or graphene). Further details about the computational methods are presented in the SI. The results for the size of the transient momentum-resolved gap and the VB shift at *T* = 1500 K are shown in Figures 4a and b, respectively. We find that the direct WS_2_ band gap at K_WS_2__ reduces by ∼11 meV (∼163 meV) for an electron density of  cm^–2^ (7 × 10^13^ cm^–2^) with a *k*-dependent variation of ∼13 meV (∼46 meV). The VB shift is found to vary between mean values of ∼2 meV and ∼70 meV in the electron density range from  cm^–2^ to 7 × 10^13^ cm^–2^ with a *k*-dependent variation between ∼6 meV and ∼35 meV. Results for *T* = 1000 K are shown in the SI in SFigure 7. Note that the calculations do not take into account capacitor-like charging shifts that occur in the transient charge-separated state. For direct comparison between theory and experiment we subtract the charging shift from the experimental data points measured for a fluence of 1.5 mJ cm^–2^ as described in detail in the SI and include them as gray dots in Figures 4a and b.

We find that the experimental data points are well reproduced by our calculations for an electron density of  cm^–2^ corresponding to the upper limit of the estimated electron–hole pair density. The experimental error bars, however, are too large to allow for an experimental verification of the theoretically predicted *k*-dependence of the transient band structure changes. In contrast to experiments, our theory allows us to disentangle various different contributions to the transient band structure changes. Based on additional data provided in the SI we conclude that (i) free-standing monolayer WS_2_ exhibits a VB shift that is roughly constant between Γ and K_WS_2__ but steeply increases at K_WS_2__ (SFigure 8). This cannot be reconciled with our experimental data in Figure 2e. (ii) WS_2_ GW contributions result in a *k*-dependent WS_2_ VB shift with three minima along the ΓK direction (SFigure 9). The average amplitudes, however, are smaller than observed in experiment. (iii) For quantitative agreement with experiment, additional graphene GdW contributions due to ultrafast hole transfer from WS_2_ to graphene need to be considered (SFigure 10). These yield a WS_2_ VB shift that is constant for the biggest part of the ΓK direction with a minimum at K_WS_2__ that appears at high carrier densities. We would like to stress that holes located in the WS_2_ monolayer itself cause much stronger renormalizations than holes located in the relatively remote graphene layer (SFigure 11). (iv) WS_2_ Hartree contributions are found to be negligible with WS_2_ VB shifts below 1 meV (SFigure 12).

In summary, we showed that WS_2_ on graphene exhibits a strong light-induced band structure renormalization that is well reproduced by our *ab initio* theory including Hartree and GW contributions of WS_2_ as well as GdW contributions of the graphene substrate. The experimental error bars, however, are too large to allow for an experimental verification of the theoretically predicted *k*-dependence of the transient band structure changes. The microscopic insights gained in this work may guide the development of future optoelectronic devices based on monolayer TMDs and their heterostructures with graphene.

## Supporting Information Available

Additional details concerning trARPES data analysis and detailed description of theory (PDF)
