# Supplementary material for: k-Resolved Ultrafast Light-Induced Band Renormalization in Monolayer WS2 on Graphene
Source: Nano Lett. 2025 Jan 8;25(3):1214–9. doi: 10.1021/acs.nanolett.4c06238 (PMC11760173; doi:10.1021/acs.nanolett.4c06238)
Supplement: Supplementary file 1 — nl4c06238_si_001.pdf [file nl4c06238_si_001.pdf]

Supporting Information for 'k-resolved ultrafast light-induced band renormalization in monolayer WS_2 on graphene'

Niklas Hofmann,[†] Alexander Steinhoff,[‡,¶] Razvan Krause,[†] Neeraj Mishra,[§,‖] Giorgio Orlandini,[§] Stiven Forti,[§] Camilla Coletti,[§,‖] Tim O. Wehling,[⊥,#] and Isabella Gierz[*,†]

†*Institute for Experimental and Applied Physics, University of Regensburg, 93040 Regensburg, Germany*

‡*Institut für Theoretische Physik, Universität Bremen, P.O. Box 330 440, 28334 Bremen, Germany*

¶*Bremen Center for Computational Materials Science, Universität Bremen, 28334 Bremen, Germany*

§*Center for Nanotechnology Innovation@NEST, Istituto Italiano di Tecnologia, Pisa, Italy*

‖*Graphene Labs, Istituto Italiano di Tecnologia, Genova, Italy*

⊥*I. Institute of Theoretical Physics, University of Hamburg, Notkestrasse 9, 22607 Hamburg, Germany*

#*The Hamburg Centre for Ultrafast Imaging, Luruper Chaussee 149, 22761 Hamburg, Germany*

E-mail: isabella.gierz@ur.de

Data Analysis

Extracting the transient band structure

In order to extract the transient position of the WS_2 valence band, the data in Fig. 1a (main manuscript) was integrated over the momentum range $\Delta k = \pm 0.04\,\text{Å}^{-1}$ around different momentum positions from $k = -0.4\,\text{Å}^{-1}$ to $k = 1.3\,\text{Å}^{-1}$. Close to the K-point, the resulting EDCs were then fitted with a constant background and three Gaussian peaks: two for the spin-split WS_2 valence band close to the K-point, and one additional peak for the valence band close to the M-point of the WS_2 flakes with a relative orientation of 30° relative to the graphene layer. Exemplary fits are presented in SFig. 1a. The following constraints were applied:

- The constant background was fixed to the value found for negative pump-probe delay.
- The energy difference between the two spin-split WS_2 valence bands was fixed to the value found for negative pump-probe delay.
- The spectral weight of the lower spin-split valence band of the flakes with 0° orientation and the M-point valence band of the flakes with 30° orientation was fixed to the value found for negative pump-probe delay.

For momentum positions between $k = -0.4\,\text{Å}^{-1}$ and $k = 1.0\,\text{Å}^{-1}$, the spin splitting of the valence band is too small to be resolved in our experiment and the valence bands of the WS_2 flakes with both orientations merge together. Thus, the band positions were extracted in the same fashion but using only one Gaussian peak.

To obtain the transient position of the WS_2 conduction band, the data in Fig. 1b (main text) was integrated over the momentum range $\Delta k = \pm 0.05\,\text{Å}^{-1}$ around $k = 1.3\,\text{Å}^{-1}$. The resulting EDCs were then fitted with a Gaussian function (see SFig. 1b).

The shift of the graphene Dirac cone was determined by integrating the data in Fig. 1a (main text) over the energy range $\Delta E = \pm 50\,\text{meV}$ around $(E - E_F) = -0.6\,\text{eV}$. The

resulting MDCs were fitted by the sum of a constant background and a Lorentzian peak (see SFig. 1c). The momentum shift obtained in this way was converted into an energy shift by multiplying with the theoretic slope of the π-band of $7\,\mathrm{eV\,\AA}$.

In order to subtract the band shift due to layer charging from the valence band shifts for Fig. 4 of the main text, the following procedure was applied for a fluence of $F = 1.5\,\mathrm{mJ\,cm^{-2}}$. It was assumed that the band shifts of valence and conduction band due to renormalization is symmetric around the center of the band gap. Then, $E_{\mathrm{gap}}(t)/2$ was added to the transient valence band position to extract the energy shift of the center of the band gap. This resulted in an upshift of $137 \pm 17\,\mathrm{meV}$, that has been subtracted from the valence band shifts of Fig. 2 of the main text.

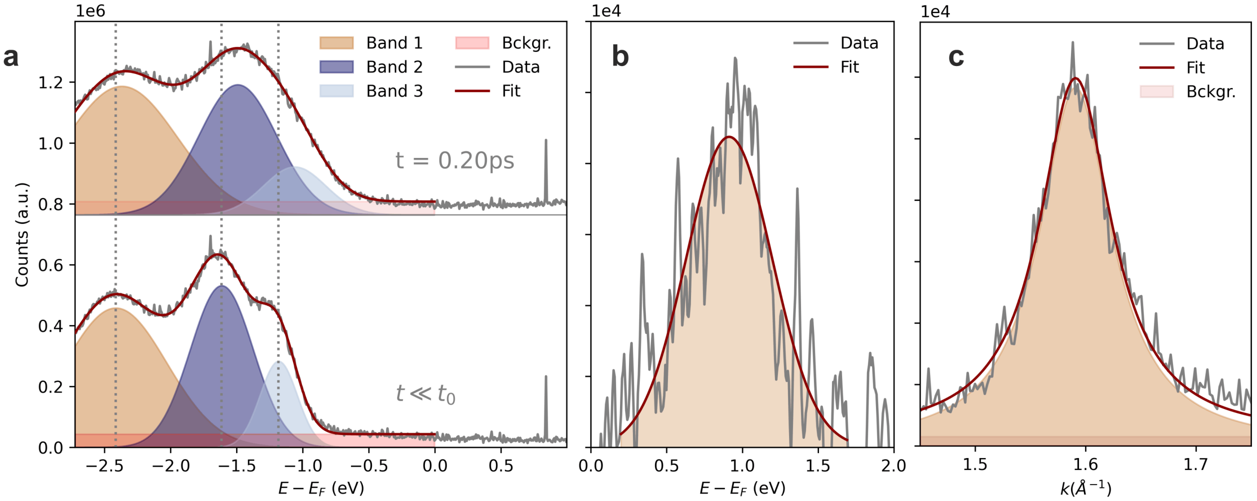

SFig. 1: **Extracting band positions.** **a)** WS_2 EDCs taken at $k = 1.3\,\mathrm{\AA}^{-1}$ for negative pump-probe delay and at $t = 0.2\,\mathrm{ps}$ fitted with a constant offset and three Gaussian peaks. The dotted vertical lines mark the peak positions for negative pump-probe delay. **b)** Pump-induced changes of the EDC through the WS_2 conduction band together with a Gaussian fit. **c)** MDC through the graphene Dirac cone at negative pump-probe delay fitted with the sum of a constant offset and a Lorentzian peak.

Exponential fit of various dynamics

For extracting rise and decay times of various pump-probe signals in the main text, an analytic fitting function was used. It was obtained by convolving the product of a step

function and an exponential decay (describing the underlying dynamics) with a Gaussian function (accounting for the finite temporal resolution):

$$f(t) = \frac{a}{2}\left(1 + \mathrm{erf}\left(\frac{(t-t_0)\tau - \frac{\mathrm{FWHM}^2}{8\ln 2}}{\sqrt{2}\tau\frac{\mathrm{FWHM}}{2\sqrt{2\ln 2}}}\right)\right)\exp\left(\frac{\frac{\mathrm{FWHM}^2}{8\ln 2} - 2(t-t_0)\tau}{2\tau^2}\right) \quad (1)$$

Here, a is the amplitude of the underlying exponential decay, erf is the error function, FWHM is the full width at half maximum of the Gaussian describing the width of the rising edge, t_0 is the center of the rising edge and τ is the decay time. This function was used to fit the dynamics showed in Figs. 2b, c and d and Fig. 3 in the main text.

Fermi-Dirac fits and transient population of Dirac cone

For extracting the transient electronic temperature and the transient chemical potential, the time- and angle-resolved photocurrent was integrated over the momentum region specified in Fig. 1 of the main text, yielding the energy-resolved occupation of the Dirac cone. These EDCs were fitted with a Fermi-Dirac distribution convolved with a Gaussian function to account for the finite energy resolution. First, the EDCs at negative delays were fitted with a fixed temperature of 294 K to obtain the width of the Gaussian. Next, the EDCs were fitted with the width of the Gaussian fixed to the value obtained for negative delays, yielding the transient electronic temperature and the transient chemical potential of the graphene layer with respect to the vacuum, $\Delta\mu^{\mathrm{vac}}$. Subsequently, the energetic shift ΔE_{gr} of the graphene π-band was obtained as described above (see SFig. 1c). The change in chemical potential $\Delta\mu$ relative to the Dirac point is then calculated as $\Delta\mu = \Delta\mu^{\mathrm{vac}} - \Delta E_{\mathrm{gr}}$. From there, one can directly extract the change of the total number of electrons in the graphene layer from

$$\Delta n_e(t) = \int_{-\infty}^{\infty} \rho(E)\left[f_{\mathrm{FD}}(E,\mu(t),T(t)) - f_{\mathrm{FD}}(E,\mu_0,T_0)\right]\,\mathrm{d}E. \quad (2)$$

Here, $\rho(E) = \frac{2A_C}{\pi}\frac{|E-E_D|}{\hbar^2 v_F^2}$ is the density of states with the energetic position of the Dirac point E_D and $A_c = \frac{3\sqrt{3}a^2}{2}$ with a lattice constant of $a = 1.42$ Å. The number of holes shown

in Fig. 3e of the main text is then given by $\Delta n_h(t) = -\Delta n_e(t)$.

Theory

Our theoretical description of atomically thin semiconductors uses the formalism of non-equilibrium Green functions in combination with ab-initio calculations for the ground-state properties. This method provides access to electronic and optical properties of semiconductors under the influence of photoexcited carriers based on material-realistic band structures and interaction matrix elements. Moreover, in a quasi-equilibrium situation frequency-dependent screening effects from a dielectric environment can be systematically taken into account. Hence we do not consider dynamics of charge-carrier populations, but focus on the time window following the optical excitation and relaxation of electrons and holes.

Band structures $\varepsilon_{\mathbf{k}}^{\lambda}$ of the optically relevant lowest conduction and highest valence bands of WS_2 are obtained from a G_0W_0-calculation as described in Refs.[1,2]. Here, λ denotes the band index, which includes spin for notational simplicity. As an interface between first-principle ground-state and excited-carrier theory we utilize a lattice Hamiltonian formulated in a localized basis of Wannier orbitals $|\alpha\rangle$, where we limit ourselves to the dominant W-d orbitals (d_{z^2}, $d_{x^2-y^2}$ and d_{xy}). Then, Bloch states are composed of Wannier obitals according to $|\psi_{\mathbf{k}}^{\lambda}\rangle = \sum_{\alpha} c_{\alpha,\mathbf{k}}^{\lambda} |\mathbf{k},\alpha\rangle$, with the coefficients $c_{\alpha,\mathbf{k}}^{\lambda}$ describing the momentum-dependent contribution of the orbital α to the Bloch band λ. The Bloch sums $|\mathbf{k},\alpha\rangle$ are connected to the localized basis via $|\mathbf{k},\alpha\rangle = \frac{1}{\sqrt{N}} \sum_{\mathbf{R}} e^{i\mathbf{k}\cdot\mathbf{R}} |\mathbf{R},\alpha\rangle$ with the number of unit cells N and lattice vectors $\mathbf{R}$. The background-screened and bare Coulomb interaction can be expressed in matrix form as $V_{\alpha\beta,\mathbf{q}}(\omega)$ and $U_{\alpha\beta,\mathbf{q}}$, respectively. The valence- and conduction-band splitting caused by spin-orbit interaction is considered along the lines of Ref. 3 and 1, including first- and second-order effects. To take into account dielectric screening by the graphene substrate, we use the *Wannier function continuum electrostatic* (WFCE) approach described in Ref. 4 that combines a continuum-electrostatic model for the screening with a localized description

of Coulomb interaction provided in Ref. 2. The dynamical screening from free charge carriers in graphene introduces a frequency dependence in the matrix elements of V.

GW self-energy with frequency-dependent background screening

On an RPA level, the Schwinger-Keldysh self-energy is[5]

$$\begin{aligned}\Sigma(1,1') &= \Sigma^{\mathrm{H}}(1,1') + \Sigma^{\mathrm{GW}}(1,1') \\ &= -i\hbar \int d2\, V(1,2) G(2,2^+) \delta(1,1') \\ &+ i\hbar G(1,1') W(1',1)\,.\end{aligned} \tag{3}$$

In a quasi-particle picture, it follows that renormalized energies are given by the self-consistency relation

$$\begin{aligned}E^{\lambda}_{\mathbf{k}} &= \varepsilon^{\lambda}_{\mathbf{k}} + \Sigma^{\mathrm{H},\lambda}_{\mathbf{k}} + \mathrm{Re}\,\Sigma^{\mathrm{GW,ret},\lambda}_{\mathbf{k}}(E^{\lambda}_{\mathbf{k}}) \\ &= \varepsilon^{\lambda}_{\mathbf{k}} + \Sigma^{\mathrm{H},\lambda}_{\mathbf{k}} + \Sigma^{\mathrm{F},\lambda}_{\mathbf{k}} + \mathrm{Re}\,\Sigma^{\mathrm{MW,ret},\lambda}_{\mathbf{k}}(E^{\lambda}_{\mathbf{k}}),\end{aligned} \tag{4}$$

where the self-energies describe photoexcited carriers in the electron-hole picture. The corresponding quasi-particle dampings are $\Gamma^{\lambda}_{\mathbf{q}} = -\mathrm{Im}\,\Sigma^{\mathrm{MW,ret},\lambda}_{\mathbf{k}}(E^{\lambda}_{\mathbf{k}})$. We have split the GW self-energy into an instantaneous Fock term and the so-called Montroll-Ward term according to the decomposition of the retarded screened Coulomb interaction matrix[5]:

$$W^{\mathrm{ret}}_{\alpha\beta,\mathbf{q}}(t) = W^{\delta}_{\alpha\beta,\mathbf{q}}\delta(t) + \theta(t)\left[W^{>}_{\alpha\beta,\mathbf{q}}(t) - W^{<}_{\alpha\beta,\mathbf{q}}(t)\right]. \tag{5}$$

Note that the Coulomb interaction W contains both, background screening from the semiconductor and its dielectric environment in the ground state and screening from photoexcited carriers[6].

The Montroll-Ward self-energy is explicitly given by:

$$\Sigma^{\mathrm{MW,ret},\lambda}_{\mathbf{k}}(\omega) = i\hbar \int_{-\infty}^{\infty} \frac{d\omega'}{2\pi} \\ \frac{1}{\mathcal{A}} \sum_{\mathbf{k}'\lambda'} \frac{(1 - f^{\lambda'}_{\mathbf{k}'}) W^{>,\lambda'\lambda\lambda'\lambda}_{\mathbf{k}'\mathbf{k}\mathbf{k}'\mathbf{k}}(\omega') + f^{\lambda'}_{\mathbf{k}'} W^{<,\lambda'\lambda\lambda'\lambda}_{\mathbf{k}'\mathbf{k}\mathbf{k}'\mathbf{k}}(\omega')}{\hbar\omega - E^{\lambda'}_{\mathbf{k}'} + i\Gamma^{\lambda'}_{\mathbf{k}'} - \hbar\omega'}, \tag{6}$$

with the Fermi distribution functions for electrons and holes $f^{\lambda}_{\mathbf{k}}$ and the crystal area $\mathcal{A}$. The band sum is limited such that electron-hole exchange is not taken into account. The plasmon propagators in Bloch representation are connected to the Coulomb matrix by:

$$W^{\gtrless,\lambda_1\lambda_2\lambda_3\lambda_4}_{\mathbf{k}_1\mathbf{k}_2\mathbf{k}_3\mathbf{k}_4}(\omega) = \sum_{\alpha,\beta} (c^{\lambda_1}_{\alpha,\mathbf{k}_1})^* (c^{\lambda_2}_{\beta,\mathbf{k}_2})^* c^{\lambda_3}_{\beta,\mathbf{k}_3} c^{\lambda_4}_{\alpha,\mathbf{k}_4} W^{\gtrless}_{\alpha\beta,\mathbf{k}_3-\mathbf{k}_2}(\omega)\,. \tag{7}$$

In quasi-equilibrium, the propagators fulfill the Kubo-Martin-Schwinger relation[5]

$$W^{>}_{\alpha\beta,\mathbf{q}}(\omega) = e^{\frac{\hbar\omega}{k_{\mathrm{B}}T}} W^{<}_{\alpha\beta,\mathbf{q}}(\omega)\,. \tag{8}$$

Combining this with the Kramers-Kronig relations for the inverse dielectric function (see below) and Eq. (5), the propagators can be expressed in terms of the retarded Coulomb interaction:

$$W^{>}_{\alpha\beta,\mathbf{q}}(\omega) = (1 + n_{\mathrm{B}}(\omega))\, 2i\, \mathrm{Im}\, W^{\mathrm{ret}}_{\alpha\beta,\mathbf{q}}(\omega), \\ W^{<}_{\alpha\beta,\mathbf{q}}(\omega) = n_{\mathrm{B}}(\omega)\, 2i\, \mathrm{Im}\, W^{\mathrm{ret}}_{\alpha\beta,\mathbf{q}}(\omega) \tag{9}$$

with the Bose distribution function $n_{\mathrm{B}}(\omega)$. The retarded Coulomb matrix is obtained using the dielectric matrix for photoexcited carriers:

$$W^{\mathrm{ret}}_{\alpha\beta,\mathbf{q}}(\omega) = \sum_{\gamma} \varepsilon^{-1,\mathrm{ret},\alpha\gamma}_{\mathrm{exc},\mathbf{q}}(\omega) V^{\mathrm{ret}}_{\gamma\beta,\mathbf{q}}(\omega)\,. \tag{10}$$

The dielectric matrix itself is given by

$$\varepsilon^{\mathrm{ret},\alpha\beta}_{\mathrm{exc},\mathbf{q}}(\omega) = \delta_{\alpha\beta} - \sum_{\gamma} V^{\alpha\gamma}_{\mathbf{q}} P^{\gamma\beta}_{\mathrm{exc},\mathbf{q}}(\omega)\,, \tag{11}$$

where we describe the polarization matrix for photoexcited carriers in the Lindhard (random-phase) approximation

$$\begin{aligned} P^{\alpha\beta}_{\mathrm{exc},\mathbf{q}}(\omega) = \frac{1}{\mathcal{A}} \sum_{\lambda,\lambda',\mathbf{k}} c^{\lambda}_{\alpha,\mathbf{k}} c^{\lambda'}_{\beta,\mathbf{k}-\mathbf{q}} \Big(c^{\lambda}_{\beta,\mathbf{k}}\Big)^{*} \Big(c^{\lambda'}_{\alpha,\mathbf{k}-\mathbf{q}}\Big)^{*} \\ \times \frac{f^{\lambda'}_{\mathbf{k}-\mathbf{q}} - f^{\lambda}_{\mathbf{k}}}{\varepsilon^{\lambda'}_{\mathbf{k}-\mathbf{q}} - \varepsilon^{\lambda}_{\mathbf{k}} + \hbar\omega + i\gamma}\,. \end{aligned} \tag{12}$$

We use a phenomenological damping $\gamma = \min(10\ \mathrm{meV},\ \hbar\omega)$ to ensure the correct analytic behavior in the static limit $\omega \to 0$. In summary, the Montroll-Ward self-energy is given by

$$\begin{aligned} \Sigma^{\mathrm{MW,ret},\lambda}_{\mathbf{k}}(\omega) = i\hbar \int_{-\infty}^{\infty} \frac{d\omega'}{2\pi} \frac{1}{\mathcal{A}} \sum_{\mathbf{k}'\lambda'} \sum_{\alpha\beta} (c^{\lambda}_{\alpha,\mathbf{k}})^{*} (c^{\lambda'}_{\beta,\mathbf{k}'})^{*} c^{\lambda}_{\beta,\mathbf{k}} c^{\lambda'}_{\alpha,\mathbf{k}'} \\ \times 2i\,\mathrm{Im}\Big\{W^{\mathrm{ret}}_{\alpha\beta,\mathbf{k}-\mathbf{k}'}(\omega')\Big\} \frac{1 - f^{\lambda'}_{\mathbf{k}'} + n_{\mathrm{B}}(\omega')}{\hbar\omega - E^{\lambda'}_{\mathbf{k}'} + i\Gamma^{\lambda'}_{\mathbf{k}'} - \hbar\omega'}\,. \end{aligned} \tag{13}$$

Ground-state renormalizations due to substrate screening

We note that even in the absence of photoexcited carriers, i.e. $P^{\alpha\beta}_{\mathrm{exc},\mathbf{q}}(\omega) = 0$ and $\varepsilon^{\mathrm{ret},\alpha\beta}_{\mathrm{exc},\mathbf{q}}(\omega) = \delta_{\alpha\beta}$, the Montroll-Ward self-energy yields a finite contribution due to the imaginary part of the frequency-dependent background-screened matrix element

$$V^{\mathrm{ret}}_{\alpha\beta,\mathbf{q}}(\omega) = \sum_{\gamma} \varepsilon^{-1,\mathrm{ret},\alpha\gamma}_{\mathrm{b},\mathbf{q}}(\omega) U_{\gamma\beta,\mathbf{q}}\,. \tag{14}$$

These quasi-particle renormalizations due to dynamical substrate screening can be interpreted as a correction to the band structure $\varepsilon^{\lambda}_{\mathbf{k}}$ of the freestanding TMD layer in the sense of a GdW calculation[7]. Using the WFCE scheme[4], the background dielectric matrix $\varepsilon^{-1,\mathrm{ret},\alpha\beta}_{\mathrm{b},\mathbf{q}}(\omega)$ is obtained from the DFT-based matrix for the freestanding WS_2 monolayer by replacing

its leading eigenvalue with a macroscopic dielectric function for the desired van der Waals heterostructure. The macroscopic dielectric function is calculated by solving Poisson's equation[8] including bulk-like dielectric functions for each layer. Due to the model character of the macroscopic dielectric function for the heterostructure, care has to be taken to properly treat the static limit of background screening. While graphene π-band screening is described by a frequency-dependent polarization (see below), screening from higher graphene bands, from the WS_2 itself, as well as from other components of the heterostructure is assumed to be static. In general, the background dielectric matrix is composed of a static and a dynamical part:

$$\varepsilon_{\mathrm{b},\mathbf{q}}^{-1,\mathrm{ret},\alpha\beta}(\omega) = \varepsilon_{\mathrm{b},\mathbf{q}}^{-1,\mathrm{stat},\alpha\beta} + \varepsilon_{\mathrm{b},\mathbf{q}}^{-1,\mathrm{dyn},\alpha\beta}(\omega)\,. \tag{15}$$

A proper dielectric function fulfilling the Kramers-Kronig relation

$$\mathrm{Re}\,\varepsilon_{\mathrm{b},\mathbf{q}}^{-1,\mathrm{ret},\alpha\beta}(\omega) = \delta_{\alpha\beta} + \mathcal{P}\int_{-\infty}^{\infty}\frac{d\omega'}{\pi}\frac{\mathrm{Im}\,\varepsilon_{\mathrm{b},\mathbf{q}}^{-1,\mathrm{ret},\alpha\beta}(\omega')}{\omega'-\omega} \tag{16}$$

would have $\varepsilon_{\mathrm{b},\mathbf{q}}^{-1,\mathrm{stat},\alpha\beta} = \delta_{\alpha\beta}$. A model dielectric function, in the simplest case describing the static limit by a constant ε_∞, leads to a violation of this behavior of the form $\varepsilon_{\mathrm{b},\mathbf{q}}^{-1,\mathrm{stat},\alpha\beta} = \delta_{\alpha\beta} + X_{\mathbf{q}}^{\alpha\beta}$. The static contribution can be extracted from the full dielectric function as

$$\varepsilon_{\mathrm{b},\mathbf{q}}^{-1,\mathrm{stat},\alpha\beta} = \mathrm{Re}\,\varepsilon_{\mathrm{b},\mathbf{q}}^{-1,\mathrm{ret},\alpha\beta}(0) - \mathcal{P}\int_{-\infty}^{\infty}\frac{d\omega'}{\pi}\frac{\mathrm{Im}\,\varepsilon_{\mathrm{b},\mathbf{q}}^{-1,\mathrm{ret},\alpha\beta}(\omega')}{\omega'}\,, \tag{17}$$

yielding a statically screened matrix element

$$V_{\mathbf{q}}^{\mathrm{stat},\alpha\beta} = \sum_{\gamma}\varepsilon_{\mathrm{b},\mathbf{q}}^{-1,\mathrm{stat},\alpha\gamma}U_{\gamma\beta,\mathbf{q}}\,. \tag{18}$$

Since the static contribution has no imaginary part, it does not contribute to the Montroll-Ward renormalization (13). While this is natural for a proper dielectric function, where

all non-trivial contributions are dynamical (i.e. $\varepsilon_{\mathrm{b},\mathbf{q}}^{-1,\mathrm{stat},\alpha\beta} = \delta_{\alpha\beta}$), we have to include the static contribution to band-structure renormalizations explicitly as an extra term. This term is derived by taking the static limit of the Montroll-Ward term (13) for $W^{\mathrm{ret}}_{\alpha\beta,\mathbf{q}}(\omega) = V^{\mathrm{ret}}_{\alpha\beta,\mathbf{q}}(\omega)$ along the lines of Ref. 6, which yields a Screened-Exchange-Coulomb-Hole self-energy. However, as discussed above, we replace $V^{\mathrm{ret}}_{\alpha\beta,\mathbf{q}}(\omega = 0)$ by the modified static matrix $V^{\mathrm{stat},\alpha\beta}_{\mathbf{q}}$ as the static limit of screened Coulomb interaction. Hence, in the Bloch representation, we have the screened matrix elements

$$V^{\mathrm{stat},\lambda\lambda'\lambda\lambda'}_{\mathbf{k}\mathbf{k}'\mathbf{k}\mathbf{k}'} = \sum_{\alpha,\beta} (c^{\lambda}_{\alpha,\mathbf{k}})^* (c^{\lambda'}_{\beta,\mathbf{k}'})^* c^{\lambda}_{\beta,\mathbf{k}} c^{\lambda'}_{\alpha,\mathbf{k}'} V^{\mathrm{stat},\alpha\beta}_{\mathbf{k}-\mathbf{k}'} \tag{19}$$

and the bare matrix elements

$$U^{\lambda\lambda'\lambda\lambda'}_{\mathbf{k}\mathbf{k}'\mathbf{k}\mathbf{k}'} = \sum_{\alpha,\beta} (c^{\lambda}_{\alpha,\mathbf{k}})^* (c^{\lambda'}_{\beta,\mathbf{k}'})^* c^{\lambda}_{\beta,\mathbf{k}} c^{\lambda'}_{\alpha,\mathbf{k}'} U^{\alpha\beta}_{\mathbf{k}-\mathbf{k}'} \,. \tag{20}$$

The resulting static self-energy is

$$\begin{aligned} \Sigma^{\mathrm{MW,stat},\lambda}_{\mathbf{k}} &= -\frac{1}{\mathcal{A}} \sum_{\mathbf{k}'\lambda'} V^{\mathrm{stat},\lambda\lambda'\lambda\lambda'}_{\mathbf{k}\mathbf{k}'\mathbf{k}'\mathbf{k}'} f^{\lambda'}_{\mathbf{k}'} \\ &\quad + \frac{1}{2\mathcal{A}} \sum_{\mathbf{k}'\lambda'} (V^{\mathrm{stat},\lambda\lambda'\lambda\lambda'}_{\mathbf{k}\mathbf{k}'\mathbf{k}'\mathbf{k}'} - U^{\lambda\lambda'\lambda\lambda'}_{\mathbf{k}\mathbf{k}'\mathbf{k}\mathbf{k}'}) \,. \end{aligned} \tag{21}$$

To avoid double-counting, we have to subtract static contributions from the ground-state ($f^{\lambda}_{\mathbf{k}} = 0$) freestanding WS_2 monolayer, which are already contained in the GW-based band structure $\varepsilon^{\lambda}_{\mathbf{k}}$:

$$\Sigma^{\mathrm{freest.},\lambda}_{\mathbf{k}} = \frac{1}{2\mathcal{A}} \sum_{\mathbf{k}'\lambda'} (V^{\mathrm{freest.},\lambda\lambda'\lambda\lambda'}_{\mathbf{k}\mathbf{k}'\mathbf{k}'\mathbf{k}'} - U^{\lambda\lambda'\lambda\lambda'}_{\mathbf{k}\mathbf{k}'\mathbf{k}\mathbf{k}'}) \,. \tag{22}$$

Note that the matrix elements U always describe bare Coulomb interaction in the freestanding monolayer. The total static self-energy is therefore given by:

$$\begin{aligned}\Sigma_{\mathbf{k}}^{\mathrm{stat},\lambda} &= \Sigma_{\mathbf{k}}^{\mathrm{MW,stat},\lambda} - \Sigma_{\mathbf{k}}^{\mathrm{freest.},\lambda} \\ &= -\frac{1}{\mathcal{A}}\sum_{\mathbf{k}'\lambda'} V_{\mathbf{kk}'\mathbf{k}'\mathbf{k}'}^{\mathrm{stat},\lambda\lambda'\lambda\lambda'} f_{\mathbf{k}'}^{\lambda'} \\ &+ \frac{1}{2\mathcal{A}}\sum_{\mathbf{k}'\lambda'}(V_{\mathbf{kk}'\mathbf{k}'\mathbf{k}'}^{\mathrm{stat},\lambda\lambda'\lambda\lambda'} - V_{\mathbf{kk}'\mathbf{kk}'}^{\mathrm{freest.},\lambda\lambda'\lambda\lambda'})\,.\end{aligned} \tag{23}$$

The second term has the form of a static GdW self-energy, with a Coulomb potential given by the difference between a strongly and a weakly screened potential.

Fock self-energy with static background screening

In the same spirit as for the ground-state contribution of screening, the static interaction $W_{\alpha\beta,\mathbf{q}}^{\delta}$ in Eq. (5) is described by the corrected Coulomb matrix $V_{\mathbf{q}}^{\mathrm{stat},\alpha\beta}$ given by Eq. (18) to obtain the Fock self-energy for excited carriers:

$$\begin{aligned}\Sigma_{\mathbf{k}}^{\mathrm{F},\lambda} = &-\frac{1}{\mathcal{A}}\sum_{\mathbf{k}'\lambda'} V_{\mathbf{kk}'\mathbf{kk}'}^{\mathrm{stat},\lambda\lambda'\lambda\lambda'} f_{\mathbf{k}'}^{\lambda'} \\ &+ \frac{1}{\mathcal{A}}\sum_{\mathbf{k}'\bar{\lambda}'} U_{\mathbf{kk}'\mathbf{kk}'}^{\lambda\bar{\lambda}'\lambda\bar{\lambda}'} f_{\mathbf{k}'}^{\bar{\lambda}'}\,.\end{aligned} \tag{24}$$

Here, the index λ' runs over bands within the same carrier species as λ, while $\bar{\lambda}'$ sums over carriers with opposite charge. As discussed in[6], electron-hole exchange is described by unscreened Coulomb matrix elements.

Hartree self-energy with frequency-dependent background screening

In the following, we derive an expression for the Hartree self-energy using the above separation of background screening into static and dynamical parts. In the Bloch basis, the self-energy

in (3) takes the form:

$$
\begin{aligned}
&\Sigma^{\mathrm{H},\lambda}_{\mathbf{k}}(t,t') \\
&= -i\hbar\frac{1}{\mathcal{A}}\sum_{\mathbf{k}'\lambda'}\int_{-\infty}^{\infty} dt_2 V^{\mathrm{ret},\lambda\lambda'\lambda'\lambda}_{\mathbf{k}\mathbf{k}'\mathbf{k}'\mathbf{k}}(t,t_2) G^{<,\lambda'}_{\mathbf{k}'}(t_2,t_2^{+})\delta(t-t') \\
&= \frac{1}{\mathcal{A}}\sum_{\mathbf{k}'\lambda}\sum_{\alpha\beta}(c^{\lambda}_{\alpha,\mathbf{k}})^{*}(c^{\lambda'}_{\beta,\mathbf{k}'})^{*}c^{\lambda'}_{\beta,\mathbf{k}'}c^{\lambda}_{\alpha,\mathbf{k}} \\
&\times\int_{-\infty}^{\infty} dt_2 V^{\mathrm{ret}}_{\alpha\beta,\mathbf{0}}(t,t_2) f^{\lambda'}_{\mathbf{k}'}(t_2)\delta(t-t')\,,
\end{aligned}
\tag{25}
$$

where we have replaced the equal-time propagator $-i\hbar G^{<}(t,t^{+})$ by single-particle occupations $f(t)$. Assuming a quasi-equilibrium state, we drop the time dependence of the latter, which leads to a time integral

$$
\int_{-\infty}^{\infty} dt_2 V^{\mathrm{ret}}_{\alpha\beta,\mathbf{0}}(t,t_2) = \sum_{\gamma}\int_{-\infty}^{\infty} dt_2 \varepsilon^{-1,\mathrm{ret},\alpha\gamma}_{\mathrm{b},\mathbf{0}}(t,t_2) U_{\gamma\beta,\mathbf{0}}\,. \tag{26}
$$

As in Eq. (15), we spilt the dielectric function into an instantaneous (static) and a retarded (dynamical) part:

$$
\begin{aligned}
&\varepsilon^{-1,\mathrm{ret},\alpha\gamma}_{\mathrm{b},\mathbf{0}}(t,t_2) = \\
&\delta(t-t_2)\varepsilon^{-1,\mathrm{stat},\alpha\gamma}_{\mathrm{b},\mathbf{0}} + \theta(t-t_2)\varepsilon^{-1,\mathrm{dyn},\alpha\gamma}_{\mathrm{b},\mathbf{0}}(t,t_2)\,.
\end{aligned}
\tag{27}
$$

Expressing the retarded part in quasi-equilibrium as

$$
\begin{aligned}
&\varepsilon^{-1,\mathrm{dyn},\alpha\gamma}_{\mathrm{b},\mathbf{0}}(\tau) \\
&= \lim_{\eta\to 0^{+}}\int_{-\infty}^{\infty}\frac{d\omega}{2\pi} e^{-i(\omega-i\eta)\tau}(\varepsilon^{-1,\mathrm{ret},\alpha\gamma}_{\mathrm{b},\mathbf{0}}(\omega) - \varepsilon^{-1,\mathrm{stat},\alpha\gamma}_{\mathrm{b},\mathbf{0}})
\end{aligned}
\tag{28}
$$

and using the modified Kramers-Kronig relation (17), we arrive at

$$
\int_{-\infty}^{\infty} dt_2 \varepsilon^{-1,\mathrm{ret},\alpha\gamma}_{\mathrm{b},\mathbf{0}}(t,t_2) = \mathrm{Re}\,\varepsilon^{-1,\mathrm{ret},\alpha\gamma}_{\mathrm{b},\mathbf{0}}(\omega=0)\,. \tag{29}
$$

Therefore, the Hartree interaction is described by the zero-frequency limit of the background-screened Coulomb potential:

$$
\begin{aligned}
\Sigma_{\mathbf{k}}^{\mathrm{H},\lambda} =& \frac{1}{\mathcal{A}} \sum_{\mathbf{k}'\lambda'} V_{\mathbf{k}\mathbf{k}'\mathbf{k}'\mathbf{k}}^{\mathrm{ret},\lambda\lambda'\lambda'\lambda}(\omega=0) f_{\mathbf{k}'}^{\lambda'} \\
&- \frac{1}{\mathcal{A}} \sum_{\mathbf{k}'\bar{\lambda}'} V_{\mathbf{k}\mathbf{k}'\mathbf{k}'\mathbf{k}}^{\mathrm{ret},\lambda\bar{\lambda}'\bar{\lambda}'\lambda}(\omega=0) f_{\mathbf{k}'}^{\bar{\lambda}'} \,.
\end{aligned} \tag{30}
$$

Hartree interaction in Wannier representation

Due to the Coulomb singularity at long wavelength ($\mathbf{q}=0$), the Hartree interaction requires a separate treatment in the Wannier representation[9]. The corresponding matrix element is given by

$$
\begin{aligned}
& V_{\mathbf{k}\mathbf{k}'\mathbf{k}'\mathbf{k}}^{\mathrm{ret},\lambda\lambda'\lambda'\lambda}(\omega=0) \\
=& \sum_{\alpha\beta} (c_{\alpha,\mathbf{k}}^{\lambda})^* (c_{\beta,\mathbf{k}'}^{\lambda'})^* c_{\beta,\mathbf{k}'}^{\lambda'} c_{\alpha,\mathbf{k}}^{\lambda} V_{\alpha\beta,\mathbf{0}}^{\mathrm{ret}}(\omega=0) \\
=& \sum_{\alpha\beta} |c_{\alpha,\mathbf{k}}^{\lambda}|^2 |c_{\beta,\mathbf{k}'}^{\lambda'}|^2 \sum_{\gamma\delta} T_{\alpha\gamma} V_{\gamma\delta,\mathbf{0}}^{\mathrm{D}}(\omega=0) T_{\delta\beta}^{\dagger} \,,
\end{aligned} \tag{31}
$$

where the columns of $T_{\alpha\gamma}$ are the eigenvectors of the Coulomb matrix (assumed to be momentum- and frequency-independent) and $V_{\gamma\delta,\mathbf{0}}^{\mathrm{D}}(\omega=0)$ is a diagonal matrix composed of the corresponding eigenvalues $V_{\gamma,\mathbf{0}}(\omega=0)$. As discussed in[4], the leading eigenvalue describes the macroscopic, or long-range properties of the Coulomb interaction, while the other eigenvalues are responsible for microscopic effects within the crystal unit cell. We can therefore decompose the Hartree self-energy as

$$
\begin{aligned}
& \Sigma_{\mathbf{k}}^{\mathrm{H},\lambda} \\
=& \frac{1}{\mathcal{A}} \sum_{\mathbf{k}'\lambda'} \Big(V_{1,\mathbf{0}}(0) \sum_{\alpha\beta} |c_{\alpha,\mathbf{k}}^{\lambda}|^2 |c_{\beta,\mathbf{k}'}^{\lambda'}|^2 T_{\alpha,1} T_{1,\beta}^{\dagger} \\
& + V_{\mathbf{k}\mathbf{k}'\mathbf{k}'\mathbf{k}}^{\mathrm{micro},\lambda\lambda'\lambda'\lambda}(0) \Big) f_{\mathbf{k}'}^{\lambda'} s^{\lambda\lambda'}
\end{aligned} \tag{32}
$$

with $s^{\lambda\lambda'}$ taking into account the sign of the Coulomb interaction term, being +1 for like charges and −1 for unlike charges. The macroscopic eigenvector has the same contribution for each of the $n_{\rm orb}$ orbitals, $\boldsymbol{T_1} = \frac{1}{\sqrt{n_{\rm orb}}}(1, 1, ..., 1)$, while the macroscopic eigenvalue is proportional to the number of orbitals: $V_{1,\mathbf{q}}(0) = n_{\rm orb}\frac{e^2}{2\varepsilon_0 q}\tilde{V}_q$ with a factor $\tilde{V}_q$ describing the non-trivial momentum dependence. Normalization of the Bloch states ($\sum_\alpha |c^\lambda_{\alpha,\mathbf{k}}|^2 = 1$) then leads to

$$\Sigma^{\rm H,\lambda}_{\mathbf{k}} = \lim_{q\to 0}\sum_{\lambda'} s^{\lambda\lambda'} n^{\lambda'} \frac{e^2}{2\varepsilon_0 q}\tilde{V}_0 + \Sigma^{\rm H,\ micro,\lambda}_{\mathbf{k}} \tag{33}$$

with the carrier density $n^\lambda = \frac{1}{\mathcal{A}}\sum_{\mathbf{k}} f^\lambda_{\mathbf{k}}$ in band λ. For a globally charge-neutral system, the macroscopic term drops out and only microscopic contributions to Hartree interaction remain. In a heterostructure exhibiting charge transfer, as in the present case of WS_2 on graphene, the individual layers become globally charged. Here, we do not attempt to quantify the corresponding charging shifts from theory but subtract them from the experimental data for direct theory-experiment comparison. Hence we calculate Hartree-type matrix elements by setting the macroscopic eigenvalue to zero before transforming the matrix element back to the Wannier representation. Note that since only the macroscopic eigenvalue is modified by environmental screening according to the WFCE scheme, Hartree interaction is not influenced by the graphene substrate but only by background screening within the WS_2 unit cell.

Frequency-dependent screening from graphene substrate

As discussed above, we describe environmental screening of Coulomb interaction in a two-dimensional layer, in particular due to a single layer of graphene below, in terms of a macroscopic dielectric function obtained from Poisson's equation for a given heterostructure[8]. This approach assumes that a single layer of graphene can be modeled as a dielectric slab with a certain effective width d_2 and an effective bulk-like dielectric function ε_2, see SFig. 2. To

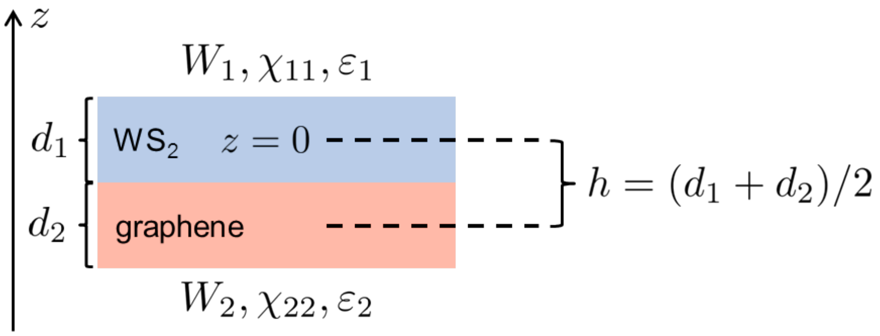

SFig. 2: Schematic of the WS_2/graphene heterostructure. W_i, χ_{ii} and ε_i denote the screened interaction, polarization and dielectric function in the respective layers. The z coordinate is centered at the WS_2-layer.

obtain accurate results, it is essential that the efficient metallic screening of graphene in the long-wavelength limit is captured by the the analytic form of ε_2. The goal of this section is to derive such an effective dielectric function from the microscopic two-dimensional polarization χ_{22} of graphene.

We start along the lines of[10] by setting up a Dyson-type equation for the screened interactions W_1 and W_2 in the WS_2 and graphene layers, which are at a distance h:

$$\varepsilon_b \begin{pmatrix} W_1 \\ W_2 \end{pmatrix} = \begin{pmatrix} V_1^{\text{ex}} \\ V_2^{\text{ex}} \end{pmatrix} + \begin{pmatrix} V_{11} & V_{12} \\ V_{21} & V_{22} \end{pmatrix} \begin{pmatrix} \chi_{11} & 0 \\ 0 & \chi_{22} \end{pmatrix} \begin{pmatrix} W_1 \\ W_2 \end{pmatrix} . \tag{34}$$

This Dyson-type approach is frequently used to describe screening in graphite, modeling the individual layers as truly two-dimensional systems with corresponding polarizations. In this sense, the approach is complementary to Poisson's equation, where each layer is effectively modeled as a three-dimensional object with bulk-like dielectric function. In Eq. (34), ε_b is a background dielectric constant, V_i^{ex} are the potentials generated by "external" charges in the layers, V_{ij} is the bare Coulomb interaction between layers i and j and χ_{ii} is the irreducible polarization of layer i. Placing a test charge in the WS_2 layer, it is $V_1^{\text{ex}} = V_q$ and $V_2^{\text{ex}} = V_q e^{-hq}$. Similary, the bare Coulomb interaction is $V_{ii} = V_q$ and $V_{12} = V_{21} = V_q e^{-hq}$.

Neglecting the polarization of WS_2, we obtain

$$W_1 = \frac{V_q}{\varepsilon_b}\Big(1 + \frac{V_q e^{-2hq}\chi_{22}}{\varepsilon_b - V_q\chi_{22}}\Big) = \frac{V_q}{\varepsilon_b}\varepsilon_q^{-1,\mathrm{Dyson}} \tag{35}$$

and

$$W_2 = \frac{V_q e^{-hq}}{\varepsilon_b - V_q\chi_{22}} \,. \tag{36}$$

Taking into account the linear behavior of the graphene π-band polarization at long wavelengths[11], $\chi_{22} \approx -\alpha q$, we obtain in the $q = 0$-limit:

$$\lim_{q\to 0} \varepsilon_q^{\mathrm{Dyson}} = \frac{\varepsilon_b + \tilde{\alpha}}{\varepsilon_b}, \quad \tilde{\alpha} = \frac{e^2}{8\hbar v_{\mathrm{F}}\varepsilon_0} \tag{37}$$

with $\tilde{\alpha} \approx 3-4$ depending on the Fermi velocity v_{F}[10–12]. Hence, unlike a two-dimensional semiconductor, a single graphene layer yields an effective dielectric constant larger than unity in the long-wavelength limit. A detailed discussion of this property is given in[11].

To draw the connection to a bulk-like effective dielectric function for graphene, we solve Poisson's equation for the heterostructure shown in SFig. 2 for $\varepsilon_1 = 1$ (corresponding to zero WS_2 polarization), which yields the following dielectric function for charges at $z = 0$:

$$\varepsilon_q^{\mathrm{Poisson}} = \frac{1 - \tilde{\varepsilon}^2 e^{-2qd_2}}{1 - \tilde{\varepsilon}^2 e^{-2qd_2} + \tilde{\varepsilon} e^{-qd_1}\left(e^{-2qd_2} - 1\right)} \tag{38}$$

with $\tilde{\varepsilon} = \frac{\varepsilon_2 - 1}{\varepsilon_2 + 1}$. More generally, Poisson's equation provides the screened potential at any position z as superposition of potentials generated by the test charge at $z = 0$ and surface charges at the graphene boundaries $j = 1, 2$ at $z_1 = -d_1/2$, $z_2 = -d_1/2 - d_2$:

$$W_q(z) = A e^{-q|z|} + \sum_j B_j e^{-q|z - z_j|} \,, \tag{39}$$

where $A = V_q/\varepsilon_b$. A straightforward calculation yields for the coefficients B_j:

$$\begin{aligned} B_1 &= \frac{A}{1-\tilde{\varepsilon}^2 e^{-2qd_2}} \tilde{\varepsilon} e^{-qd_1/2} (\tilde{\varepsilon} e^{-2qd_2} - 1)\,, \\ B_2 &= \frac{A}{1-\tilde{\varepsilon}^2 e^{-2qd_2}} \tilde{\varepsilon} e^{-q(d_1/2+d_2)} (1-\tilde{\varepsilon})\,. \end{aligned} \tag{40}$$

Hence we obtain for the total surface charge potential of graphene:

$$\begin{aligned} B &= B_1 + B_2 \\ &= \frac{A}{1-\tilde{\varepsilon}^2 e^{-2qd_2}} \tilde{\varepsilon} e^{-qd_1/2} \left(\tilde{\varepsilon} e^{-2qd_2} - 1 + e^{-qd_2} - \tilde{\varepsilon} e^{-qd_2} \right) \\ &= A \tilde{\varepsilon} e^{-qd_1/2} \frac{\left(1+\tilde{\varepsilon} e^{-qd_2}\right)\left(e^{-qd_2}-1\right)}{1-\tilde{\varepsilon}^2 e^{-2qd_2}} \\ &= A \tilde{\varepsilon} e^{-qd_1/2} \frac{e^{-qd_2}-1}{1-\tilde{\varepsilon} e^{-qd_2}}\,. \end{aligned} \tag{41}$$

The limit of an infinitely thin graphene slab is calculated by letting $d_2 \to 0$ and $d_1/2 \to h$:

$$B \to A \tilde{\varepsilon} e^{-qh} \frac{qd_2}{\tilde{\varepsilon}(1-qd_2)-1}\,. \tag{42}$$

Similar to Eq. (39), we can interpret the screened potentials W_1 and W_2 from the Dyson-type equation (Eqs. (35) and (36)) as superpositions of a test charge at $z = 0$ and a graphene surface charge at $z = -h$:

$$W_q^{\mathrm{D}}(z) = A_{\mathrm{D}} e^{-q|z|} + B_{\mathrm{D}} e^{-q|z+h|}\,. \tag{43}$$

By setting $W_1 = W_q^{\mathrm{D}}(z=0)$ and $W_2 = W_q^{\mathrm{D}}(z=-h)$, we find

$$\begin{aligned} A_{\mathrm{D}} &= \frac{V_q}{\varepsilon_b}, \\ B_{\mathrm{D}} &= \left(\frac{V_q}{\varepsilon_b}\right)^2 e^{-qh} \frac{\chi_{22}}{1-V_q/\varepsilon_b \chi_{22}} = \left(\frac{V_q}{\varepsilon_b}\right)^2 e^{-qh} \tilde{\chi}_{22}\,. \end{aligned} \tag{44}$$

While χ_{22} is the *irreducible* polarization describing the density response to the total electric field within the graphene layer, the quantity

$$\begin{aligned}\tilde{\chi}_{22} &= \chi_{22}(1 - V_q/\varepsilon_b\chi_{22})^{-1} \\ &= \chi_{22} + \chi_{22}V_q/\varepsilon_b\chi_{22} + \chi_{22}V_q/\varepsilon_b\chi_{22}V_q/\varepsilon_b\chi_{22} + ...\end{aligned} \tag{45}$$

is the *reducible* polarization responding to the external field alone. Identifying the surface charge coefficient B (42) from Poisson's equation with the coefficient B_{D} (44) from Dyson's equation for a vacuum background ($\varepsilon_b = 1$) allows to derive the effective dielectric function for graphene in terms of the graphene polarization χ_{22}. We find

$$\tilde{\varepsilon} = \frac{\varepsilon_2 - 1}{\varepsilon_2 + 1} = \frac{V_q\tilde{\chi}_{22}}{V_q\tilde{\chi}_{22} - qd_2(1 + V_q\tilde{\chi}_{22})}\,, \tag{46}$$

from which it follows that

$$\begin{aligned}\varepsilon_2 &= 1 - \frac{2}{qd_2}\frac{V_q\tilde{\chi}_{22}}{1 + V_q\tilde{\chi}_{22}} \\ &= 1 - \frac{2}{qd_2}V_q\chi_{22}\,.\end{aligned} \tag{47}$$

Note that by choosing a vacuum environment for the heterostructure and neglecting the polarization of the TMD itself, we obtained an effective bulk-like dielectric function describing the pristine screening effects of a single graphene layer.

Finally, we introduce the microscopic irreducible polarization of graphene π-bands according to Ref. 10:

$$\begin{aligned}\chi^{\mathrm{gr}}_{\mathbf{q}}(\omega) &= 2\frac{1}{\mathcal{A}}\sum_{b,b'=c,v}\sum_{\mathbf{k}}\frac{1}{4}\Big(1 + q^2/36\Big)^{-6} \\ &\times\left|1 \pm \frac{H_{12}(\mathbf{k}+\mathbf{q})H^*_{12}(\mathbf{k})}{|H_{12}(\mathbf{k}+\mathbf{q})H^*_{12}(\mathbf{k})|}\right|\frac{f^{b'}_{\mathbf{k}+\mathbf{q}} - f^b_{\mathbf{k}}}{\varepsilon^{b'}_{\mathbf{k}+\mathbf{q}} - \varepsilon^b_{\mathbf{k}} + \hbar\omega + i\gamma}\,,\end{aligned} \tag{48}$$

where we choose $\mathbf{q} = (q, 0)$ for simplicity. $+$ and $-$, respectively, correspond to intraband and interband excitations. $f^b_{\mathbf{k}}$ denote Fermi distribution functions at a given temperature and chemical potential. The dispersion is given by

$$\varepsilon^{c,v}_{\mathbf{k}} = \pm\gamma_0\Big[1 + 4\cos(\sqrt{3}ak_y/2)\cos(ak_x/2) + 4\cos^2(ak_x/2)\Big]^{1/2} \tag{49}$$

and $\gamma_0 = 2/\sqrt{3}\hbar v_{\mathrm{F}}/a$ with the DFT-based Fermi velocity $v_{\mathrm{F}} = 950$ nm ps^{-1} [12] and the lattice constant $a = 0.246$ nm[10] ($\sqrt{3}$ times the C-C bond length). The matrix elements H_{12} are given by

$$H_{12}(\mathbf{k}) = -\gamma_0\Big[e^{-ik_y a/\sqrt{3}} + 2e^{ik_y a/\sqrt{3}/2}\cos(ak_x/2)\Big]^{1/2}. \tag{50}$$

We use a damping $\gamma = \min(10\text{ meV}, \hbar\omega)$ to ensure the correct analytic behavior in the static limit $\omega \to 0$. Note that $\chi^{\mathrm{gr}}_{\mathbf{q}}(\omega)$ does not contain screening contributions from higher graphene bands, which are sometimes included as an additional (high-frequency) constant[10,13]. Here, we describe the additional screening contribution by a model polarization $\chi^{\mathrm{gr},\infty}_{\mathbf{q}} = (1 - \varepsilon^{\mathrm{gr},\infty}_{\mathbf{q}})/V_q$, with a dielectric function $\varepsilon^{\mathrm{gr},\infty}_{\mathbf{q}}$ that has been adjusted to first-principle cRPA calculations[14]:

$$\begin{aligned}\varepsilon^{\mathrm{gr},\infty}_{\mathbf{q}} &= \varepsilon_\infty \frac{\varepsilon_\infty + 1 - (\varepsilon_\infty - 1)e^{-qd_\infty}}{\varepsilon_\infty + 1 + (\varepsilon_\infty - 1)e^{-qd_\infty}}\,, \\ \varepsilon_\infty &= 2.4\,, d_\infty = 0.28\,\mathrm{nm}\,.\end{aligned} \tag{51}$$

In the following, we use the function ε_2 from Eq. (47) with a standard 2-d Coulomb potential $V_q = V^{2d}_q = \frac{e^2}{2\varepsilon_0 q}$ as dielectric function of graphene in Poisson's equation. To this end, we explicitly set $\chi_{22} = \chi^{\mathrm{gr}}_{\mathbf{q}}(\omega) + \chi^{\mathrm{gr},\infty}_{\mathbf{q}}$ as irreducible polarization:

$$\varepsilon_2 = \varepsilon^{\mathrm{gr}}_{\mathbf{q}}(\omega) = 1 - \frac{2}{qd_{\mathrm{gr}}}\frac{V^{2d}_q(\chi^{\mathrm{gr}}_{\mathbf{q}}(\omega) + \chi^{\mathrm{gr},\infty}_{\mathbf{q}})}{1 - V^{2d}_q(\chi^{\mathrm{gr}}_{\mathbf{q}}(\omega) + \chi^{\mathrm{gr},\infty}_{\mathbf{q}})}\,. \tag{52}$$

As graphene layer thickness d_2, we use the inter-layer distance in graphite $d_{\mathrm{gr}} = 0.335$ nm[4].

Results for the pristine heterostructure

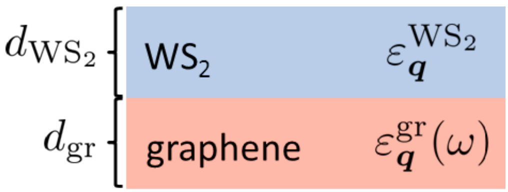

SFig. 3: Schematic of the WS_2/graphene heterostructure.

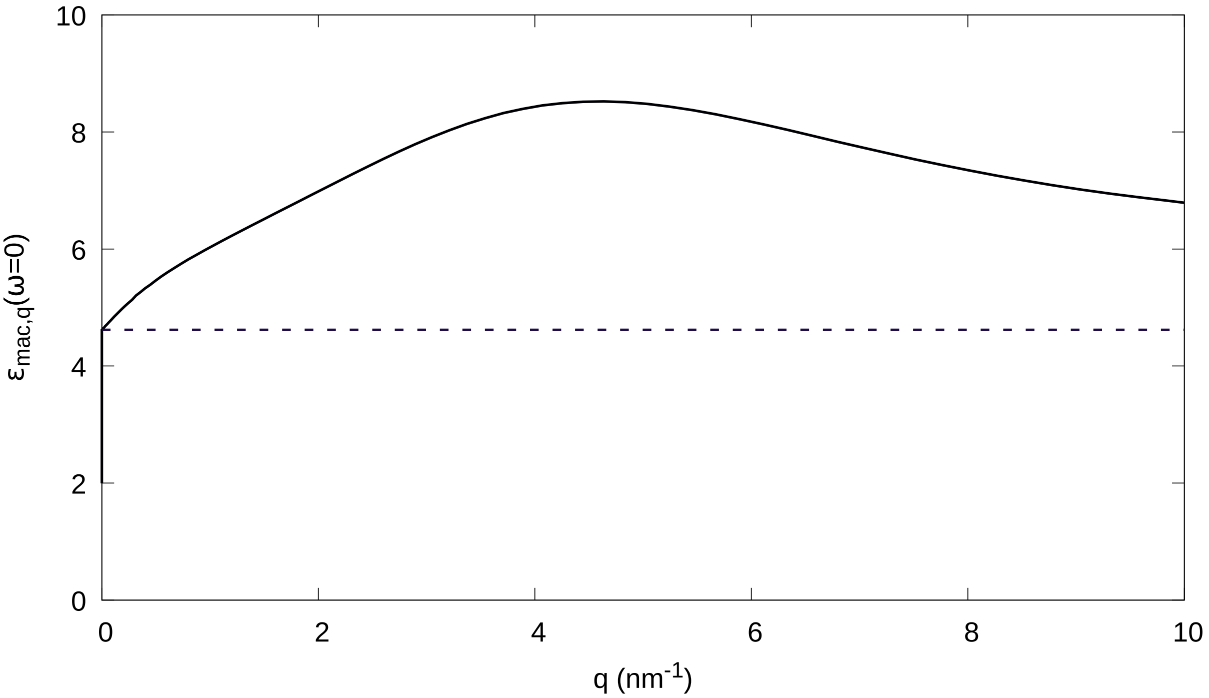

SFig. 4: Static limit of the macroscopic dielectric function for carriers in a WS_2 monolayer embedded in the heterostructure from SFig. 3 (solid line). For comparison, the analytic long-wavelength limit $1 + \tilde{\alpha}$ with $\tilde{\alpha}$ from Eq. (37) is shown as a dashed line.

To benchmark our approach to quasi-particle renormalizations induced by screening from a graphene substrate, we evaluate the dynamical Montroll-Ward self-energy, Eq. (13), for a fixed quasi-particle broadening of 10 meV in the absence of photoexcited carriers, i.e. $\varepsilon^{\mathrm{ret},\alpha\beta}_{\mathrm{exc},\mathbf{q}}(\omega) = \delta_{\alpha\beta}$, $f^{\lambda}_{\mathbf{k}} = 0$ and $E^{\lambda}_{\mathbf{k}} = \varepsilon^{\lambda}_{\mathbf{k}}$. We add the corresponding static GdW contribution

given by Eq. (23). The result is a correction to the band structure of freestanding monolayer WS_2:

$$\Sigma_{\mathbf{k}}^{\mathrm{GdW,gr},\lambda} = \Sigma_{\mathbf{k}}^{\mathrm{MW,ret},\lambda}\Big|_{f_{\mathbf{k}}^{\lambda}=0}(\omega = \frac{\varepsilon_{\mathbf{k}}^{\lambda}}{\hbar}) + \Sigma_{\mathbf{k}}^{\mathrm{stat},\lambda}\Big|_{f_{\mathbf{k}}^{\lambda}=0} . \tag{53}$$

As discussed above, screening from the dielectric environment is taken into account via the WFCE approach, replacing the leading eigenvalue of the microscopic background dielectric matrix of monolayer WS_2 by a macroscopic dielectric function for the given heterostructure. The macroscopic dielectric function is given by the Poisson solution for the heterostructure shown in SFig. 3 with $\varepsilon_{\mathbf{q}}^{\mathrm{WS_2}}$ and $d_{\mathrm{WS_2}}$ as parametrized in Ref. 2 and $\varepsilon_{\mathbf{q}}^{\mathrm{gr}}(\omega)$ from Eq. (52). We assume zero graphene doping. In SFig. 4, the static limit of the macroscopic dielectric function is shown. Remarkably, the long-wavelength limit is larger than 1 due to efficient quasi-metallic screening from graphene.

The momentum-dependent band shift resulting from Eq. (53) is shown in SFig. 5. The renormalizations exhibit a weak band dependence, but a pronounced momentum dependence between Γ and K. In the electron-hole picture, both renormalizations are negative, which means that they yield a band-gap shrinkage of about $440 - 480$ meV in total relative to the freestanding WS_2 monolayer.

Photoexcited heterostructure inlcuding substrate

To describe screening in the full heterostructure including a substrate, we derive a macroscopic dielectric function of the WS_2/Gr/SiC-heterostructure, see SFig. 6. We model the pump-probe experiment assuming a sufficiently long delay after the pump pulse so that relaxation of carriers has taken place. Hence electrons and holes in the heterostructure assume a quasi-equilibrium distribution with an effective temperature that may be significantly higher than the lattice temperature. We assume that recombination of electron-hole pairs takes

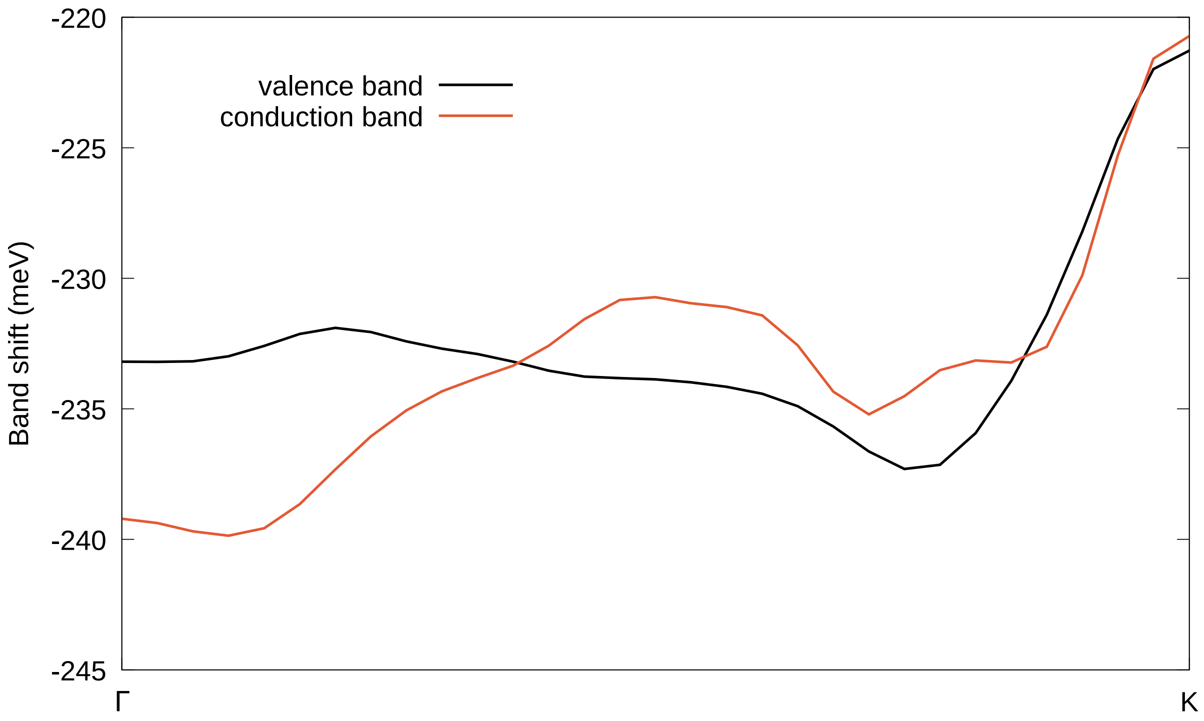

SFig. 5: Quasi-particle band shift of the highest valence and lowest conduction bands of monolayer WS_2 on an undoped graphene substrate between Γ and K points in the absence of photoexcited carriers. The shifts are given in the electron-hole picture, i.e. both are leading to a band-gap shrinkage compared to freestanding monolayer WS_2.

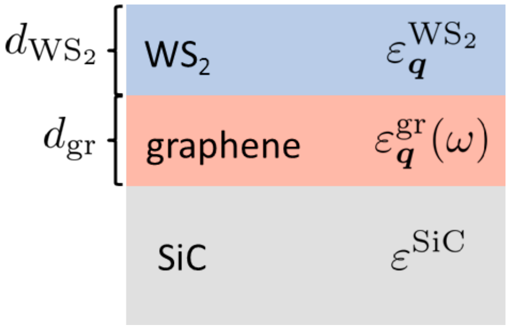

SFig. 6: Schematic of the WS_2/graphene/SiC heterostructure.

place on longer time scales. Prior to excitation, no carriers are excited in WS_2, while graphene is hole-doped with a Fermi energy $E_F = -300$ meV, corresponding to a hole density $n_h^{gr,0} = (E_F/\hbar/v_F)^2/\pi = 7.3 \times 10^{12}\text{cm}^{-2}$. Graphene carriers are assumed to be at room temperature ($T = 300$ K). After optical excitation of WS_2 creating an electron-hole pair density $n_e^{WS_2}$, most of the holes are transferred to graphene as discussed in the main text.

We assume that 10 percent of the holes remain in the WS_2 layer, i.e. $n_h^{WS_2} = 0.1 \times n_e^{WS_2}$. Correspondingly, assuming a 50% coverage of the graphene flakes by WS_2, the hole density in graphene after carrier relaxation amounts to $n_h^{gr} = n_h^{gr,0} + 0.5 \times 0.9 \times n_e^{WS_2}$. We assume that carriers in WS_2 and graphene have a common effective temperature.

First of all, we correct the freestanding WS_2 band structure before the optical excitation by adding static GdW corrections due to screening from the heterostructure shown in SFig. 6. To this end, we replace $\varepsilon_{\mathbf{k}}^{\lambda}$ by $\varepsilon_{\mathbf{k}}^{\lambda} + \Sigma_{\mathbf{k}}^{\mathrm{stat},\lambda}\big|_{f_{\mathbf{k}}^{\lambda}=0}$ using Eq. (23). Here, $\varepsilon_{\mathbf{q}}^{WS_2}$ and d_{WS_2} are parametrized as in Ref. 2, $\varepsilon_{\mathbf{q}}^{gr}(\omega)$ is taken from Eq. (52) and $\varepsilon^{\mathrm{SiC}} = 6.5$[15] is the dielectric constant of the semi-infinite substrate. Carriers in graphene are distributed as before the pump pulse.

The pump-induced shift of WS_2 quasi-particle energies consists of two parts: (i) Hartree-Fock and Montroll-Ward renormalizations due to WS_2 carriers and (ii) screening-induced renormalizations due to graphene carriers. Here, graphene carriers are treated as "background" contribution to screening via the substrate dielectric function, while only carriers in WS_2 contribute to excited-carrier screening.
Renormalizations due to WS_2 carriers are computed by evaluating Eq. (4) with the self-energies (13), (24) and (30) self-consistently. Background screening is given by the doped graphene without excited carriers and excited-carrier screening due to WS_2 carriers is described by a dielectric matrix in RPA as given by Eqs. (11) and (12):

$$\Sigma_{\mathbf{k}}^{WS_2,\lambda} = \Sigma_{\mathbf{k},\mathrm{gr\ doped}}^{\mathrm{H},\lambda} + \Sigma_{\mathbf{k},\mathrm{gr\ doped}}^{\mathrm{F},\lambda} + \Sigma_{\mathbf{k},\mathrm{gr\ doped}}^{\mathrm{MW,ret},\lambda}(\omega = \frac{E_{\mathbf{k}}^{\lambda}}{\hbar}) \,. \tag{54}$$

The Montroll-Ward term $\Sigma_{\mathbf{k},\mathrm{gr\ doped}}^{\mathrm{MW,ret},\lambda}$ would contain dynamical background screening even in the absence of excited carriers in WS_2 (for $\varepsilon_{\mathrm{exc},\mathbf{q}}^{\mathrm{ret},\alpha\beta}(\omega) = \delta_{\alpha\beta}$, see Eq. (11)) and therefore already induce some renormalizations. Hence we subtract a corresponding term without

excited carriers to get a purely pump-induced shift due to WS_2 carriers:

$$\Sigma_{\mathbf{k}}^{\mathrm{WS_2},\Delta,\lambda} = \Sigma_{\mathbf{k}}^{\mathrm{WS_2},\lambda} - \Sigma_{\mathbf{k}}^{\mathrm{WS_2},\lambda}\Big|_{n^{\mathrm{WS_2}}=0} . \tag{55}$$

The pump-induced shift of quasi-particle energies due to graphene carriers is simulated by computing the sum of dynamical (13) and static (23) Montroll-Ward self-energies for the graphene carrier distributions before and after the pulse. For the WS_2 carrier populations $f_{\mathbf{k}}^{\lambda}$ and quasi-particle energies $E_{\mathbf{k}}^{\lambda}$, we use the values obtained from the self-consistent calculation of excited-carrier self-energies. For the excited-carrier screening, we again use $\varepsilon_{\mathrm{exc},\mathbf{q}}^{\mathrm{ret},\alpha\beta}(\omega) = \delta_{\alpha\beta}$ to capture just the background effects of graphene. In this sense, different carrier distributions in graphene before and after the pulse induce different dielectric screening experienced by the WS_2 layer, leading to a differential quasi-particle renormalization. Hence, we calculate the graphene-induced GdW shift as

$$\begin{aligned}\Sigma_{\mathbf{k}}^{\mathrm{GdW,gr},\lambda} &= \Sigma_{\mathbf{k},\mathrm{gr\ exc.}}^{\mathrm{MW,ret},\lambda}\Big|_{P_{\mathrm{exc}}=0}(\omega = \frac{E_{\mathbf{k}}^{\lambda}}{\hbar}) \\ &- \Sigma_{\mathbf{k},\mathrm{gr\ doped}}^{\mathrm{MW,ret},\lambda}\Big|_{P_{\mathrm{exc}}=0}(\omega = \frac{E_{\mathbf{k}}^{\lambda}}{\hbar}) \\ &+ \Sigma_{\mathbf{k},\mathrm{gr\ exc.}}^{\mathrm{stat},\lambda} - \Sigma_{\mathbf{k},\mathrm{gr\ doped}}^{\mathrm{stat},\lambda} .\end{aligned} \tag{56}$$

Band-structure enormalizations in the photoexcited heterostructure for $T = 1000$ K

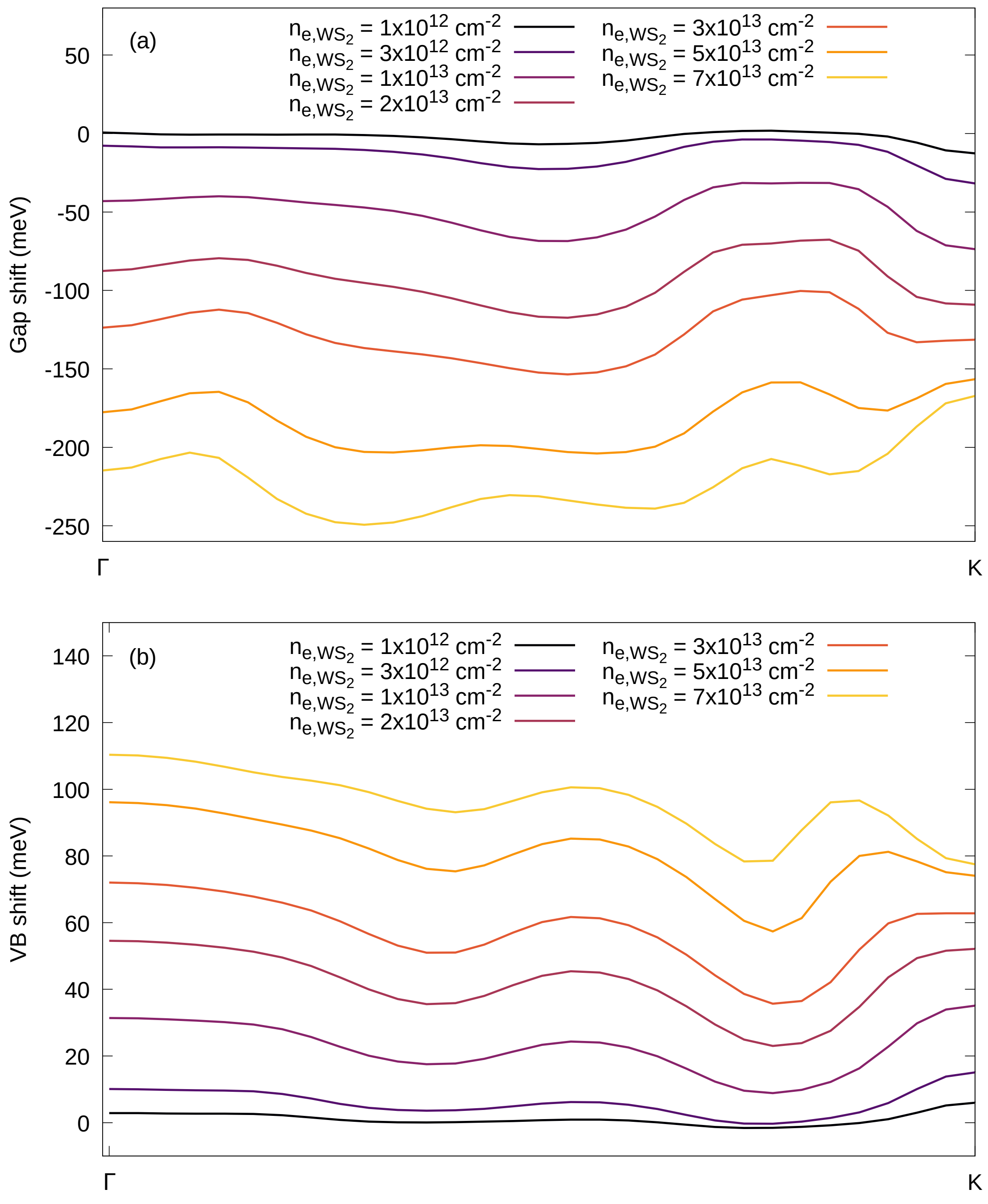

SFig. 7: Calculated k-resolved transient band gap (a) and quasi-particle shifts of WS_2 VB (b) for different carrier densities for a carrier temperature of $T = 1000\,\mathrm{K}$.

Monolayer WS_2 results

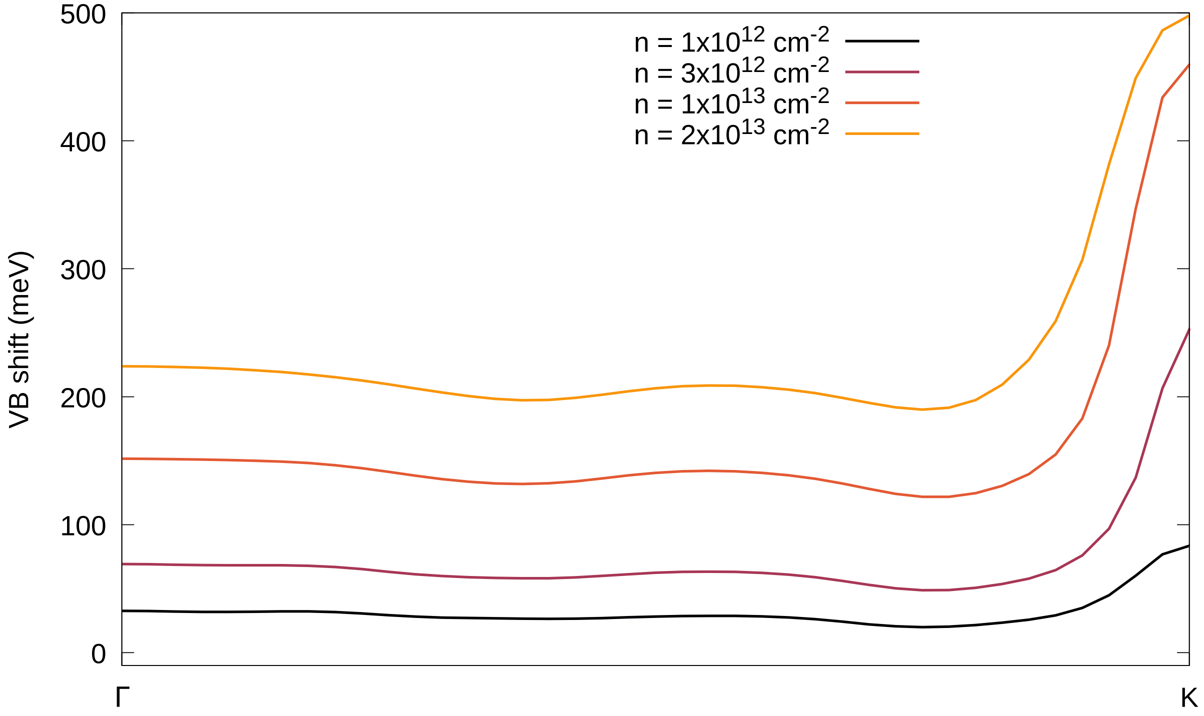

SFig. 8: Quasi-particle shifts of valence band (= minus hole energy) in monolayer WS_2 for different carrier densities at $T = 1000$ K.

Different contributions to valence-band renormalizations in photoexcited heterostructure

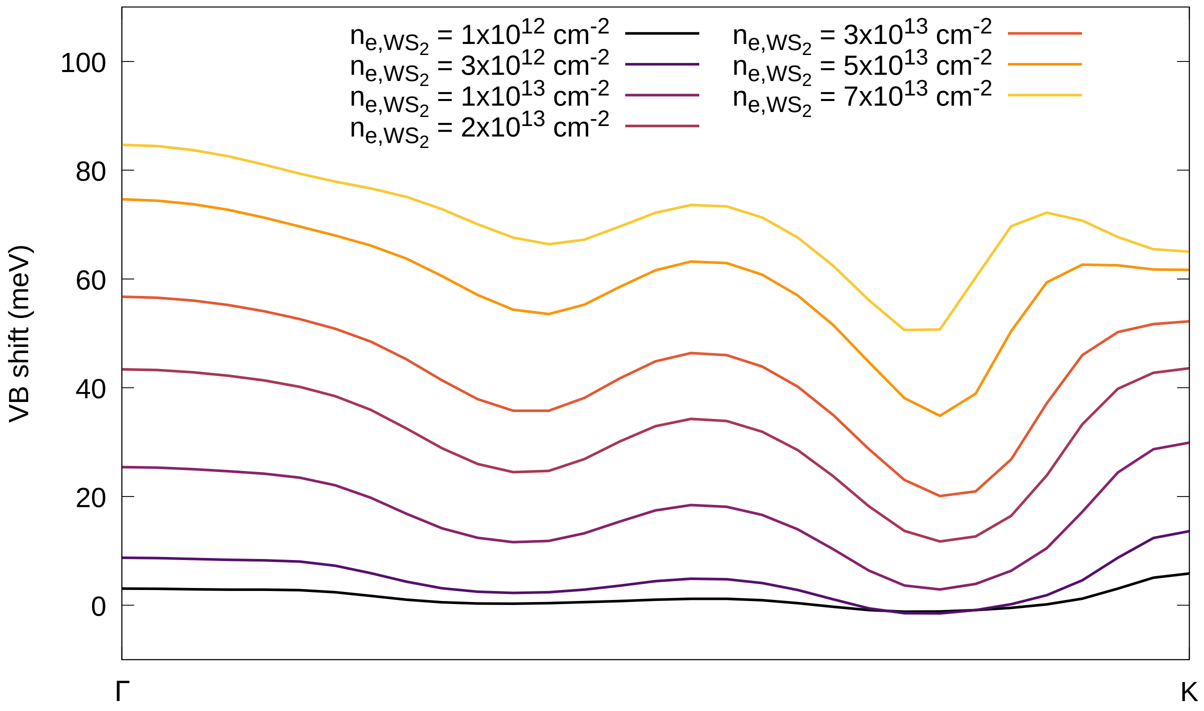

SFig. 9: Only WS_2 GW contributions to valence-band shifts for different carrier densities at $T = 1000$ K (sum of Fock and Montroll-Ward terms in Eq. (55)).

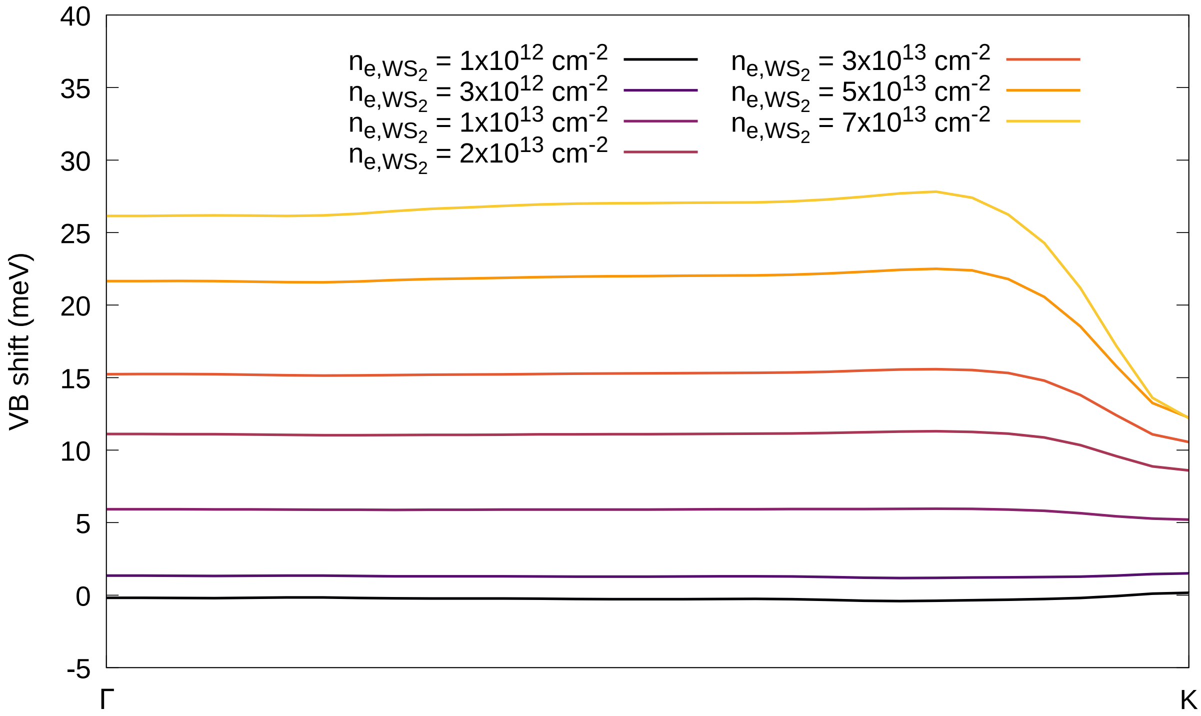

SFig. 10: Only graphene GdW contributions to valence-band shifts for different carrier densities at $T = 1000$ K (Eq. (56)).

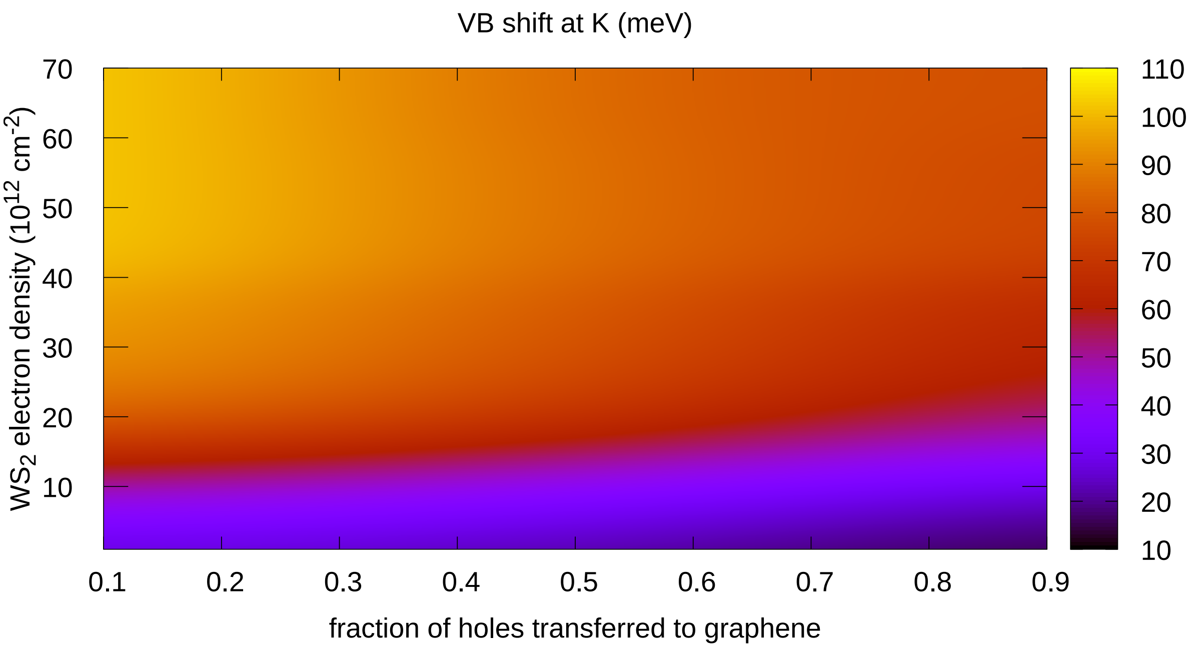

SFig. 11: Dependence of WS_2 VB shift at K in meV including all above-mentioned contributions as a function of WS_2 electron density and fraction of holes transferred to graphene at $T = 1000$ K.

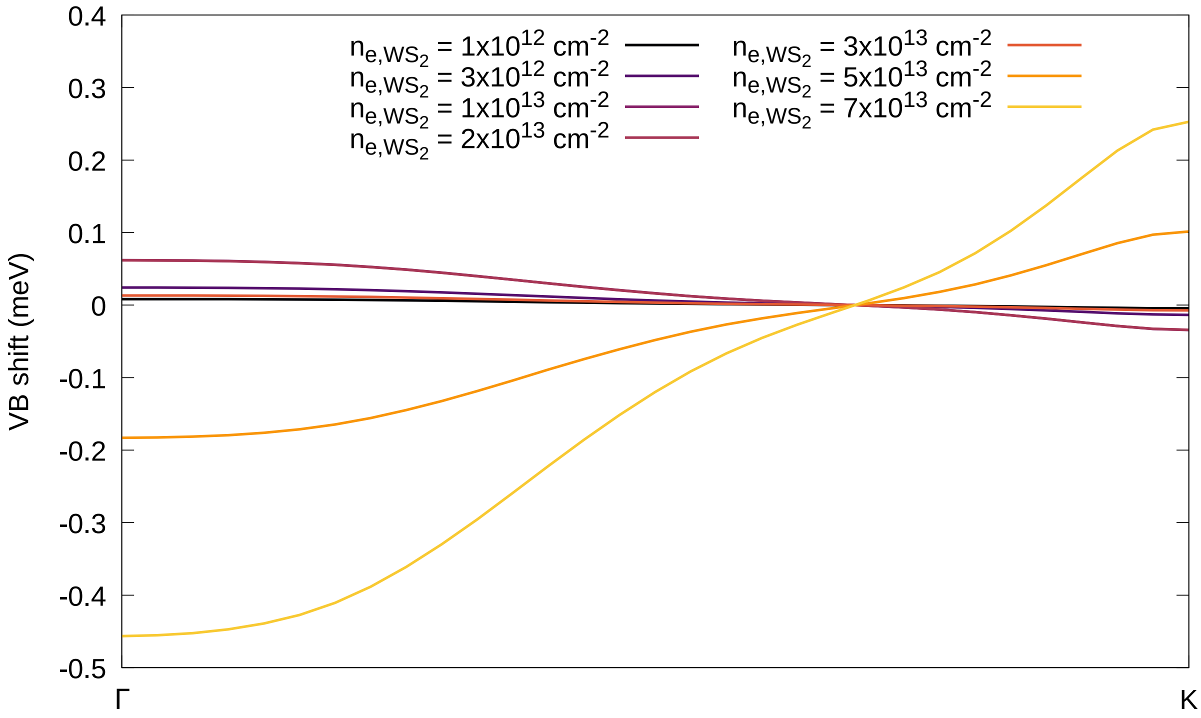

SFig. 12: Only WS_2 Hartree contributions to valence-band shifts for different carrier densities at $T = 1000$ K (Hartree term in Eq. (55)).

References

(1) Steinhoff, A.; Rösner, M.; Jahnke, F.; Wehling, T. O.; Gies, C. Influence of Excited Carriers on the Optical and Electronic Properties of MoS2. *Nano Letters* **2014**, *14*, 3743–3748.

(2) Steinhoff, A.; Florian, M.; Rösner, M.; Schönhoff, G.; Wehling, T. O.; Jahnke, F. Exciton fission in monolayer transition metal dichalcogenide semiconductors. *Nature Communications* **2017**, *8*, 1166.

(3) Liu, G.-B.; Shan, W.-Y.; Yao, Y.; Yao, W.; Xiao, D. Three-band tight-binding model for monolayers of group-VIB transition metal dichalcogenides. *Physical Review B* **2013**, *88*, 085433.

(4) Rösner, M.; Şaşioğlu, E.; Friedrich, C.; Blügel, S.; Wehling, T. O. Wannier function approach to realistic Coulomb interactions in layered materials and heterostructures. *Physical Review B* **2015**, *92*, 085102.

(5) Kremp, D.; Schlanges, M.; Kraeft, W.-D. *Quantum Statistics of Nonideal Plasmas*; Springer, Berlin, 2005.

(6) Erben, D.; Steinhoff, A.; Gies, C.; Schönhoff, G.; Wehling, T. O.; Jahnke, F. Excitation-induced transition to indirect band gaps in atomically thin transition-metal dichalcogenide semiconductors. *Physical Review B* **2018**, *98*, 035434.

(7) Rohlfing, M. Electronic excitations from a perturbative $\text{LDA}+GdW$ approach. *Physical Review B* **2010**, *82*, 205127.

(8) Florian, M.; Hartmann, M.; Steinhoff, A.; Klein, J.; Holleitner, A. W.; Finley, J. J.; Wehling, T. O.; Kaniber, M.; Gies, C. The Dielectric Impact of Layer Distances on Exciton and Trion Binding Energies in van der Waals Heterostructures. *Nano Letters* **2018**, *18*, 2725–2732.

(9) Schobert, A.; Berges, J.; van Loon, E. G. C. P.; Sentef, M. A.; Brener, S.; Rossi, M.; Wehling, T. O. Ab initio electron-lattice downfolding: Potential energy landscapes, anharmonicity, and molecular dynamics in charge density wave materials. *SciPost Physics* **2024**, *16*, 046.

(10) Lin, M. F.; Huang, C. S.; Chuu, D. S. Plasmons in graphite and stage-1 graphite intercalation compounds. *Physical Review B* **1997**, *55*, 13961–13971, Publisher: American Physical Society.

(11) Hwang, E. H.; Das Sarma, S. Dielectric function, screening, and plasmons in two-dimensional graphene. *Physical Review B* **2007**, *75*, 205418, Publisher: American Physical Society.

(12) Trevisanutto, P. E.; Giorgetti, C.; Reining, L.; Ladisa, M.; Olevano, V. Ab Initio \$GW\$ Many-Body Effects in Graphene. *Physical Review Letters* **2008**, *101*, 226405, Publisher: American Physical Society.

(13) Shung, K. W. K. Dielectric function and plasmon structure of stage-1 intercalated graphite. *Physical Review B* **1986**, *34*, 979–993, Publisher: American Physical Society.

(14) Wehling, T. O.; Şaşıoğlu, E.; Friedrich, C.; Lichtenstein, A. I.; Katsnelson, M. I.; Blügel, S. Strength of Effective Coulomb Interactions in Graphene and Graphite. *Physical Review Letters* **2011**, *106*, 236805.

(15) Patrick, L.; Choyke, W. J. Static Dielectric Constant of SiC. *Physical Review B* **1970**, *2*, 2255–2256, Publisher: American Physical Society.
